# CHD4 and SMYD1 repress common transcriptional programs in the developing heart

**DOI:** 10.1242/dev.202505

**Published:** 2024-05-03

**Authors:** Wei Shi, Lauren K. Wasson, Kerry M. Dorr, Zachary L. Robbe, Caralynn M. Wilczewski, Austin J. Hepperla, Ian J. Davis, Christine E. Seidman, Jonathan G. Seidman, Frank L. Conlon

**Affiliations:** ^1^ Department of Biology and Genetics, McAllister Heart Institute, UNC-Chapel Hill, Chapel Hill, NC 27599, USA; ^2^ Department of Genetics, Harvard Medical School, Boston, MA 02115, USA; ^3^ Department of Medicine and Howard Hughes Medical Institute, Chevy Chase, MD 20815, USA; ^4^ Lineberger Comprehensive Cancer Center, University of North Carolina at Chapel Hill, Chapel Hill, NC 27599, USA; ^5^ Division of Cardiovascular Medicine, Brigham and Women's Hospital, Boston, MA 02115, USA

**Keywords:** SMYD1, CHD4, Transcription, Chromatin accessibility, Mouse

## Abstract

Regulation of chromatin states is essential for proper temporal and spatial gene expression. Chromatin states are modulated by remodeling complexes composed of components that have enzymatic activities. CHD4 is the catalytic core of the nucleosome remodeling and deacetylase (NuRD) complex, which represses gene transcription. However, it remains to be determined how CHD4, a ubiquitous enzyme that remodels chromatin structure, functions in cardiomyocytes to maintain heart development. In particular, whether other proteins besides the NuRD components interact with CHD4 in the heart is controversial. Using quantitative proteomics, we identified that CHD4 interacts with SMYD1, a striated muscle-restricted histone methyltransferase that is essential for cardiomyocyte differentiation and cardiac morphogenesis. Comprehensive transcriptomic and chromatin accessibility studies of *Smyd1* and *Chd4* null embryonic mouse hearts revealed that SMYD1 and CHD4 repress a group of common genes and pathways involved in glycolysis, response to hypoxia, and angiogenesis. Our study reveals a mechanism by which CHD4 functions during heart development, and a previously uncharacterized mechanism regarding how SMYD1 represses cardiac transcription in the developing heart.

## Teaser

**Summary:** Comprehensive transcriptomic and chromatin accessibility studies of *Smyd1* and *Chd4* null embryonic mouse hearts reveal that SMYD1 and CHD4 repress a group of common genes and pathways in developing hearts.

## INTRODUCTION

Understanding the molecular mechanisms and associated genetics of cardiac development is essential for improving the treatment of congenital heart defects, a leading cause of infant mortality. Numerous signaling pathways precisely regulate early cardiogenesis at the transcriptional level (Bruneau, 2002; Buckingham et al., 2005). Dynamic cardiac gene expression is controlled epigenetically by large, multi-component complexes that modify chromatin structure and modulate the accessibility of regulatory sequences to transcriptional activators and repressors (Akerberg and Pu, 2020; Bruneau et al., 2019). The nucleosome remodeling and deacetylase (NuRD) complex is a prominent chromatin-modifying complex that mediates gene repression. The NuRD complex is composed of an ATP-dependent chromodomain helicase DNA binding protein 3/4 (CHD3/4), a histone deacetylase (HDAC1/2), a metastasis-associated protein (MTA1/2/3), a retinoblastoma-binding protein (RBBP4/7), and a GATAD2A/2B subunit. By the combined action of its histone deacetylase and ATP-dependent chromatin remodeling helicase, the NuRD complex modulates chromatin states at the target genes (Marhold et al., 2004; Wade et al., 1999, 1998; Xue et al., 1998; Zhang et al., 1998). The NuRD complex is essential for numerous developmental events, including heart development. Genetic studies with mice have demonstrated that cardiomyocyte-conditional *Chd4* null (*Chd4-CMko*) mutants die at mid-gestation; further, the absence of CHD4-mediated gene repression leads to misexpression of fast skeletal and smooth muscle myofibril isoforms, cardiac sarcomere malformation, and early embryonic lethality (Gomez-Del Arco et al., 2016; Wilczewski et al., 2018). We recently showed that a missense mutation in CHD4 results in congenital heart defects in both humans and mice (Shi et al., 2023), and we identified that CHD4 is recruited by the core cardiac transcription factors GATA4, NKX2-5 and TBX5 to specific cardiac gene loci in embryonic hearts (Robbe et al., 2022; Waldron et al., 2016).

SMYD1 (formerly BOP), an evolutionarily conserved SET and MYND domain-containing histone methyltransferase, is restricted to striated muscles and is essential for early cardiac development (Gottlieb et al., 2002; Phan et al., 2005; Rasmussen et al., 2015; Tracy et al., 2018; Wang et al., 2021). Global knockdown of *smyd1a* and *smyd1b* in zebrafish disrupts myofibril formation and leads to an absence of beating in the heart (Tan et al., 2006). Conventional null *Smyd1* (*Smyd1-KO*) mice die *in utero* at embryonic day (E) 10.5 owing to disrupted maturation of cardiomyocytes and malformation of the right ventricle (Gottlieb et al., 2002). In addition, SMYD1 is required for maintaining cardiomyocyte proliferation during embryonic heart development. Its loss leads to linked stress responses that signal ensuing lethality (Oka et al., 2020; Rasmussen et al., 2015; Warren et al., 2018). Many mechanisms have been reported for how SMYD1 functions in the developing heart. First, SMYD1 functions as a repressor by the interaction of its MYND domain with co-repressors HDAC1-3, NCoR (NCOR1) and SMRT (NCOR2) (Gottlieb et al., 2002; Sims et al., 2002). Second, SMYD1 functions as a transcriptional activator by catalyzing trimethylation of H3K4, a histone modification associated with transcriptionally active loci (Tan et al., 2006). Lastly, in skeletal and heart muscle, SMYD1 interacts and colocalizes with skNAC (NACA), the muscle-restricted isoform of the DNA-binding protein nascent polypeptide-associated complex (Berkholz et al., 2014; Park et al., 2010). Thus, SMYD1 regulates gene transcription by different mechanisms. However, the SMYD1-associated gene expression profile and how SMYD1 mediates chromatin accessibility in developing hearts have not been established; the SMYD1 interactome in cardiomyocytes has yet to be determined.

To determine how CHD4 regulates heart development and to identify other CHD4-interacting proteins besides the NuRD components in the heart, we performed immune-affinity tandem mass spectrometry (IP-MS/MS) assays to compile a CHD4 cardiac interactome in mouse embryonic heart at E10.5 (Robbe et al., 2022). Surprisingly, we found that CHD4 interacts with the essential cardiac histone methyltransferase SMYD1 in the developing heart. We used co-immunoprecipitation (co-IP), proximity ligation assay (PLA) and surface plasmon resonance (SPR) to confirm that the CHD4–SMYD1 interaction was direct. By performing transcriptomic analyses (RNA-seq) and transposase-accessible chromatin by deep sequencing (ATAC-seq) on both *Smyd1-KO* and *Chd4-CMko* embryonic hearts, we identified a specific group of genes and pathways that were repressed by both SMYD1 and CHD4, including those involved in glycolysis, response to hypoxia, and angiogenesis. Our study revealed a mechanism by which CHD4 functions in striated muscles, and we also identified how SMYD1 represses cardiac transcription in the developing heart.

## RESULTS

### CHD4 interacts with methyltransferase SMYD1 in the heart

Previously, we performed mass spectrometry (MS) analysis of IP-MS/MS-purified CHD4 complexes obtained from cardiac nuclei derived from embryonic mouse hearts at E10.5 (Robbe et al., 2022). This analysis defined the CHD4 endogenous cardiac interactome at the time when CHD4 is essential for cardiac development (Gomez-Del Arco et al., 2016; Wilczewski et al., 2018). We recovered CHD4 from cardiac tissue at 57% coverage of a theoretical maximum of 86.6% with a trypsin digest (Fig. S1A,B). In the current study, we used an unbiased gene ontology (GO)-based bioinformatics classification to screen the functions of proteins associated with CHD4. This analysis defined a subset of candidate interactions. In agreement with published reports, functional network analysis showed that CHD4 interacts with MTA1/2/3, RBBP4/7, GATAD2A/2B and HDAC1/2 (Tables S1, S3), all components of the NuRD transcriptional repression complex (Wade et al., 1999, 1998; Xue et al., 1998; Zhang et al., 1998).

**Fig. 1.**
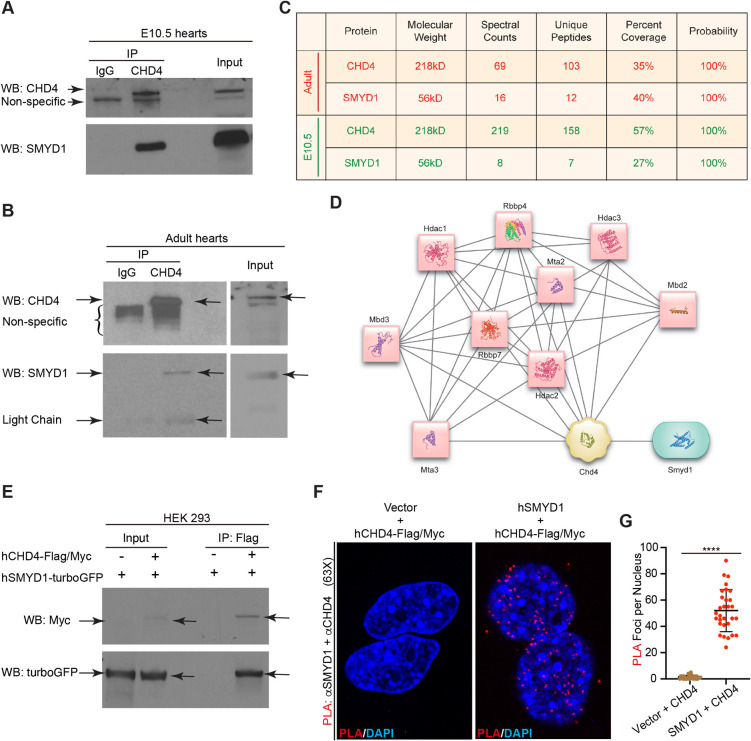
**SMYD1 interacts with CHD4 in the heart.** (A) Immuno-isolation of endogenous CHD4 from embryonic hearts shows interaction with endogenous SMYD1. E10.5 WT CD1 hearts (minimum 28 hearts per IP, three separate biological replicates) were used. (B) Immuno-isolation of endogenous CHD4 from adult cardiac nucleus shows interaction with endogenous SMYD1. Three adult (6-week-old) CD1 mouse hearts were pooled and homogenized. The lysate was split in half, and IgG- and CHD4-IP were performed on each of the half lysates. Two independent experiments were repeated. (C) Peptide coverage of CHD4 and SMYD1 proteins of CHD4 IP/MS quantification. (D) Protein–protein interaction network centered on CHD4 generated by STRING with high confidence using proteins in the CHD4-IP interactome. (E) Flag/Myc-tagged, full-length human CHD4 (hCHD4-Flag/Myc) and turboGFP-tagged, full-length human SMYD1 (hSMYD1-turboGFP) were transfected into HEK-293 cells. IP of exogenous CHD4 shows interaction with exogenous SMYD1. Three independent experiments were repeated. (F) Constructs of Flag/Myc-tagged, full-length human CHD4 and full-length human SMYD1 were transfected into HEK-293 cells. Proximity ligation assay (PLA) was performed using anti-SMYD1 and anti-CHD4 antibodies with a PLA assay kit, and the nuclei were stained with DAPI. CHD4–SMYD1 interaction occurs in the nucleus, as shown by the red foci. (G) Quantification of the red foci in the nucleus in each condition. Data were obtained from 30 cells (from three independent replicates) per condition and are shown as mean±s.e.m. ****P<0.0001 (Welch's *t*-test).

Our analysis of the CHD4 cardiac interactome further revealed that CHD4 interacts with the methyltransferase SMYD1 (Fig. 1A, Fig. S1C, Tables S1, S3). The CHD4–SMYD1 interaction was of special interest because SMYD1 expression is specific to cardiac and skeletal muscles (Phan et al., 2005; Rasmussen et al., 2015; Tracy et al., 2018), and mice homozygous null for *Smyd1* (*Smyd1-KO*) die of heart defects by E10.5 (Gottlieb et al., 2002), the same developmental stage that requires CHD4. To determine whether the CHD4–SMYD1 interaction also occurs in adult hearts, we repeated IP-MS/MS analysis with adult cardiac nuclei and recovered CHD4 at 35% and SMYD1 at 40% (Fig. 1B-D). Together, these results demonstrate that CHD4 and SMYD1 interact in both embryonic and adult hearts.

We next performed reciprocal immunoprecipitation (IP) assays with transfected HEK-293 cells and confirmed that both mouse and human orthologs of CHD4 and SMYD1 interact (Fig. 1E, Fig. S2A-C). To determine the location of the CHD4–SMYD1 interaction in the cells, we conducted PLAs in transfected HEK-293 cells (Jalili et al., 2018; Robbe et al., 2022). In agreement with our IP-MS/MS results from isolated cardiac nuclei, we found that CHD4 and SMYD1 interact in the nucleus (Fig. 1F,G, Fig. S2D,E). We further examined the interaction of endogenous CHD4 and SMYD1 in isolated embryonic cardiac cells by performing a PLA with co-staining of tropomyosin (a marker of cardiomyocytes), and we determined that the CHD4–SMYD1 interaction specifically occurs in the nucleus of tropomyosin-positive cells (cardiomyocytes) (Fig. S2F-J).

As the catalytic subunit of the NuRD complex, CHD4 is composed of a large C-terminal ATPase/helicase module (CHD4-C) and a set of N-terminal structural-functional domains that include highly conserved tandem plant homeodomain fingers and two chromodomains (CHD4-N). Studies have established that CHD4-C is the main region responsible for protein interaction (Allen et al., 2013; Reid et al., 2023). For example, notable enrichment of NuRD subunits was seen with CHD4–C compared with CHD4–N. ADNP (a subunit of the ChAHP complex) and the transcription repressor NAB2 also interact with CHD4 through the CHD4-C (Sharifi Tabar et al., 2022; Srinivasan et al., 2006). Therefore, we generated purified protein corresponding to the C-terminal portion of CHD4 to test the ability of CHD4-C to interact with SMYD1 and to determine whether the interaction between CHD4 and SMYD1 is direct. Using an SPR (Nguyen et al., 2015), we measured the affinity of CHD4-C for individual peptides tiled across full-length SMYD1 (Fig. S3A). From these studies, we identified a single 30-residue peptide from SMYD1 (SMYD1-Region 13: LSYLQAYEEASHYARRMVDGYMKLYHNNA) for which CDH4 had an affinity >10^9^ (Fig. S3B). This peptide is 90% conserved between mice and humans, and molecular modeling showed that the sequence maps to SMYD1 helix X and helix XI (Fig. S3C). Because only the purified CHD4 protein and the SMYD1 peptides were present in the SPRs, our results demonstrate that CHD4 and SMYD1 can interact directly.

### CHD4 and SMYD1 share embryonic cardiac interactomes

**Fig. 2.**
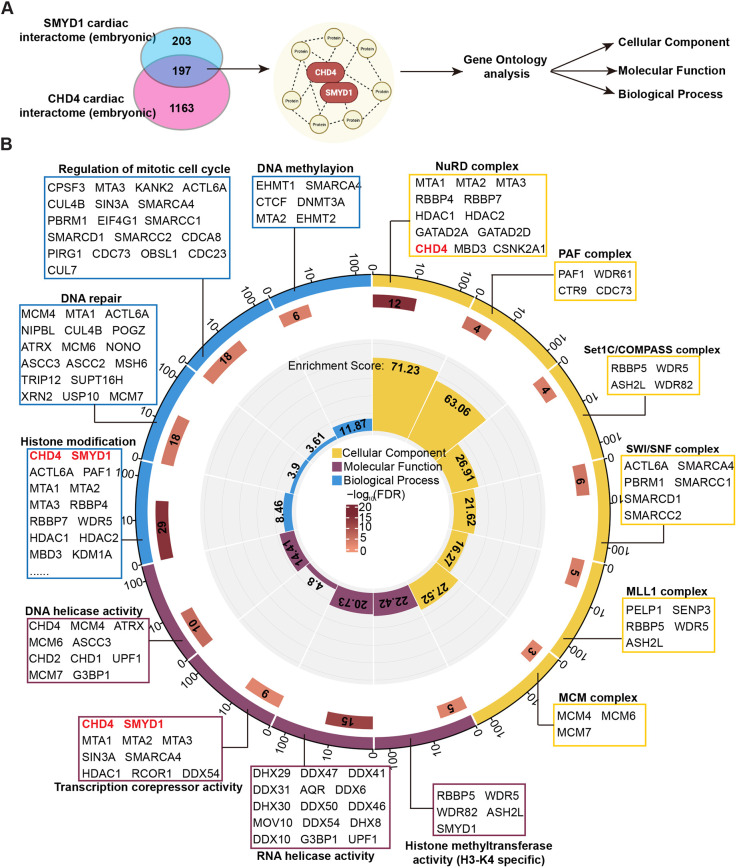
**CHD4 and SMYD1 cardiac interactomes.** (A) CHD4 and SMYD1 embryonic cardiac interactomes and overlapping analyses. (B) GO analyses for CHD4 and SMYD1 cardiac interactome-shared proteins.

To determine whether CHD4 and SMYD1 exist in the same cardiac interactome in the developing heart, we performed MS analysis of IP-MS/MS-purified SMYD1 complexes obtained from cardiac nuclei derived from embryonic mouse hearts at E9.5 when SMYD1 is essential for heart development in mice (Gottlieb et al., 2002). Four-hundred proteins were identified in the SMYD1 cardiac interactome, of which 197 proteins were shared by CHD4 and SMYD1 embryonic cardiac interactomes (Fig. 2A, Tables S1-S3).

Unbiased GO-based bioinformatics classification was performed to characterize the functions of the shared 197 proteins. Cellular component analysis showed that proteins shared by the CHD4 and SMYD1 cardiac interactomes include components of the NuRD complex (i.e. MTA1/2/3, HDAC1/2, RBBP4/7, GATAD2A/2D, MBD3 and CSNK2A1), suggesting that SMYD1 interacts with CHD4 in the context of the NuRD complex in the developing hearts. Shared proteins are also involved in other protein complexes, including the PAF [i.e. PAF1, WDR61 (SKIC8)], COMPASS (i.e. RBBP5, WDR5), SWI/SNF (i.e. ACTL6A, SMARCA4), MLL1 (i.e. PELP1, SENP3), and MCM (i.e. MCM4/6/7) complexes. Biological processes and molecular function analyses revealed that both CHD4 and SMYD1 are related to histone modification, transcription repression, DNA repair, and regulation of the mitotic cell cycle (Fig. 2B, Tables S1-S3). All these biological events are closely related to early heart development. These analyses demonstrate that CHD4 and SMYD1 could exert similar functions through the same protein complexes.

### SMYD1 activates and represses gene expression in the developing heart

**Fig. 3.**
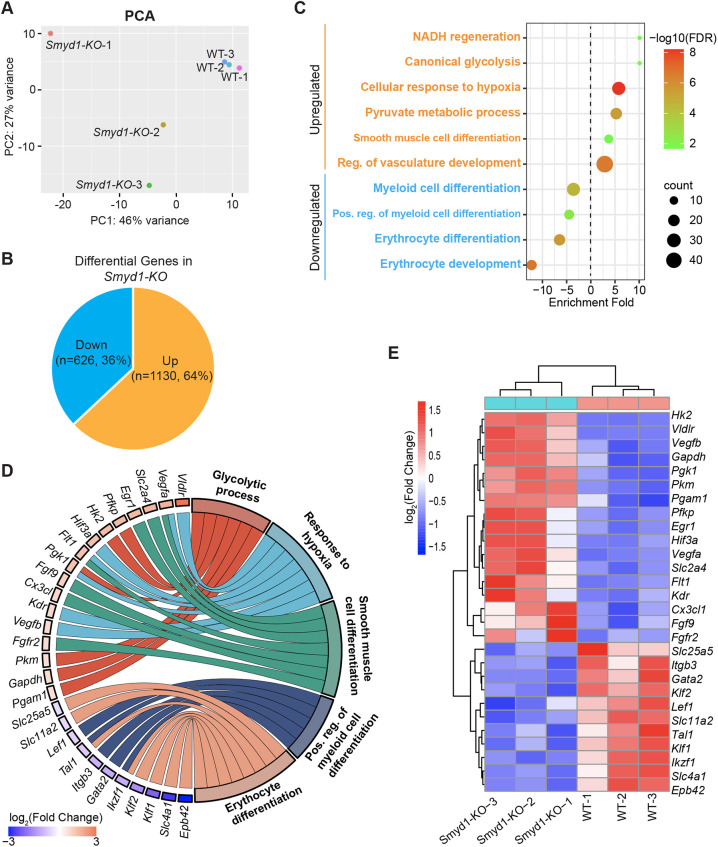
**Transcriptomes of embryonic *Smyd1-KO* hearts.** (A) Principal component analysis (PCA) plot of gene expression data from RNA-seq of *n*=4 replicates for each genotype at E9.5. (B) In E9.5 *Smyd1-KO* hearts, 626 genes were downregulated and 1130 genes were upregulated (adjusted *P*≤0.05 and log_2_ fold change≥±0.5). (C) Hallmark pathway enrichment of differentially expressed genes in E9.5 *Smyd1-KO* hearts. Sizes of circles represent gene counts, and the color bar represents −log_10_(FDR-adjusted *P*-value). (D) The circular plot of representative differentially expressed genes in *Smyd1-KO* hearts simultaneously presents a detailed view of the relations between expression changes (left semicircle perimeter) and enriched biological processes (right semicircle perimeter). Color bar represents log_2_ fold change of gene expression originally from the RNA-seq differential gene expression analysis. (E) Heatmap of the genes shown in D. Color bar represents normalized log_2_ fold change of gene expression.

SMYD1 has two alternate functions in remodeling chromatin and regulating transcription (Tracy et al., 2018; Wang et al., 2021). SMYD1 is considered to be a transcriptional repressor because it recruits histone deacetylase (HDAC) (Gottlieb et al., 2002), and SMYD1 also functions as a transcriptional activator by tri-methylating H3K4 at promoter regions (Oka et al., 2020; Wang et al., 2021; Warren et al., 2018). Embryos homozygous for a mutated *Smyd1* allele (*Smyd1-KO*) had disrupted maturation of ventricular cardiomyocytes, formation of the right ventricle was dampened, and the embryos died by E10.5 (Gottlieb et al., 2002; Phan et al., 2005; Rasmussen et al., 2015; Sims et al., 2002). To define comprehensively the molecular mechanism by which SMYD1 functions in heart development, we performed transcriptomic analysis (RNA-seq) on E9.5 wild-type (WT) and *Smyd1-KO* hearts (Fig. 3A); we focused on stage E9.5 to reflect the state immediately before the stage in which cardiac defects occurred in *Smyd1* null hearts. By comparing transcript abundances in the presence (WT) or absence (*Smyd1-KO*) of *Smyd1*, we identified 1756 differentially expressed genes at stage E9.5 [adjusted *P*<0.05, log_2_(fold change)≥±0.5] (Fig. 3B, Table S4). In agreement with the dual functions of SMYD1 in transcription, in the absence of *Smyd1*, 1130 genes (64% of 1756) were upregulated and 626 genes (36% of 1756) were downregulated (Fig. 3B).

We performed GO analyses to investigate the functions and pathways of differentially expressed genes in *Smyd1* null hearts. Surprisingly, the most significant over-represented pathways associated with upregulated genes (e.g. *Vegfa*, *Hk2*, *Fgf9*) transcriptionally regulated by SMYD1 were glycolysis, response to hypoxia, and smooth muscle cell differentiation (Fig. 3C-E). Downregulated genes (e.g. *Lef1*, *Ikzf1*, *Klf1*) were enriched in myeloid cell and erythrocyte differentiation (Fig. 3C-E). These results suggest that SMYD1 regulates metabolism and cell differentiation in the developing hearts.

### SMYD1 and CHD4 repress the common cardiac gene programs

**Fig. 4.**
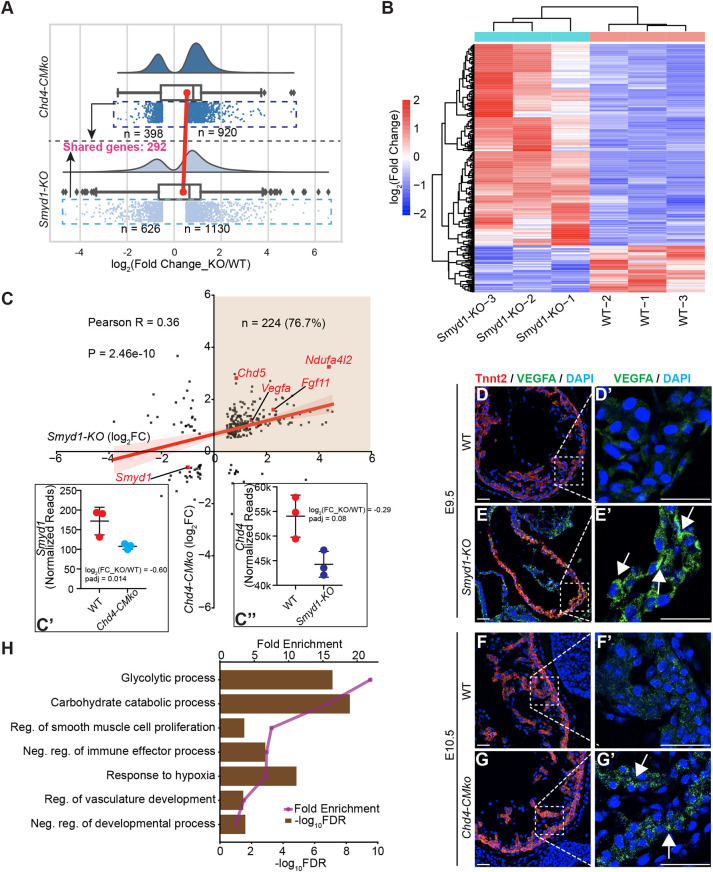
**SMYD1 and CHD4 regulate a common subset of genes in embryonic hearts.** (A) Raincloud plots for *Chd4-CMko* and *Smyd1-KO* differential genes. Dots in the dashed boxes represent individual data point (‘rain’), and the half-violins represent data distribution (‘cloud’). The lower (Q1) and upper (Q3) quartiles in both boxes are 25th and 75th percentile, respectively, and the interquartile range (IQR) showing the middle 50% of values defines each box. The whiskers are Q1−1.5*IQR and Q3+1.5*IQR on each side of the box, respectively. The black vertical lines within each box show the medians of each data set, and the red line connects the means of each data set. The skewness of the *Chd4-CMko* data set and the *Smyd1-KO* data set are −0.1484 and −0.05142, respectively. (B) Heatmap of the 292 shared genes in A, clustered based on *Smyd1-KO* RNA-seq datasets. Color bar represents normalized log_2_ fold change of gene expression. (C) Dot plot of differentially expressed genes in *Smyd1-KO* (*x*-axis) log_2_ fold change (FC) against that in *Chd4-CMko* (*y*-axis) log_2_ FC showing a positive correlation between the two mutant hearts at overall mRNA expression level (Pearson R=0.35; *P*=2.46e−10); 224 genes (76.7% of 292) were upregulated in both *Smyd1-KO* and *Chd4-CMko* hearts. (C′,C″) Normalized reads of *Smyd1* and *Chd4* genes from the corresponding RNA-seq. Mean±s.e.m., *n*=3 per genotype. (D-G′) Representative images of immunofluorescence [cardiac troponin T (Tnnt2, VEGFA and DAPI]-stained sections from E9.5 *Smyd1-KO* (and WT) and E10.5 *Chd4-CMko* (and WT) mouse hearts. Arrows indicate positive VEGFA staining. Scale bars: 50 µm. (H) PANTHER GO over-representation test in biological processes terms for the 224 shared upregulated genes from C. Bar length represents −log_10_ (FDR), and the purple line represents the fold enrichment of each GO term.

Having established the interaction of SMYD1 and CHD4 in the developing heart, we then investigated whether SMYD1 and CHD4 mediate similar or the same cardiac gene expression programs. Two hundred and ninety-two differentially expressed genes are shared in *Smyd1-KO* and *Chd4-CMko* hearts (Fig. 4A-C, Table S5), including *Smyd1*, which was significantly downregulated in the hearts of both mutant lines (Fig. 4C′). A similar expression pattern was observed for the *Chd4* gene, but the downregulation of this gene was not significant in *Smyd1-KO* hearts (Fig. 4C″). Among these shared genes, 224 (76.7% of 292) were upregulated in both *Smyd1-KO* and *Chd4-CMko* hearts (Fig. 4C). As an example, at both transcript and protein levels, the expression of VEGFA was much higher in both *Smyd1-KO* and *Chd4-CMko* hearts compared with their WT littermates (Fig. 4C-G′). We then performed GO term analyses, and as observed in the *Smyd1-KO* hearts, these 224 shared upregulated genes were over-represented in the glycolytic process, response to hypoxia, and regulation of smooth muscle cell differentiation and development (Fig. 4H). These results strongly indicate that SMYD1 and CHD4 function by repressing common gene programs and pathways to regulate heart development.

### Chromatin accessibility in *Smyd1-KO* hearts

**Fig. 5.**
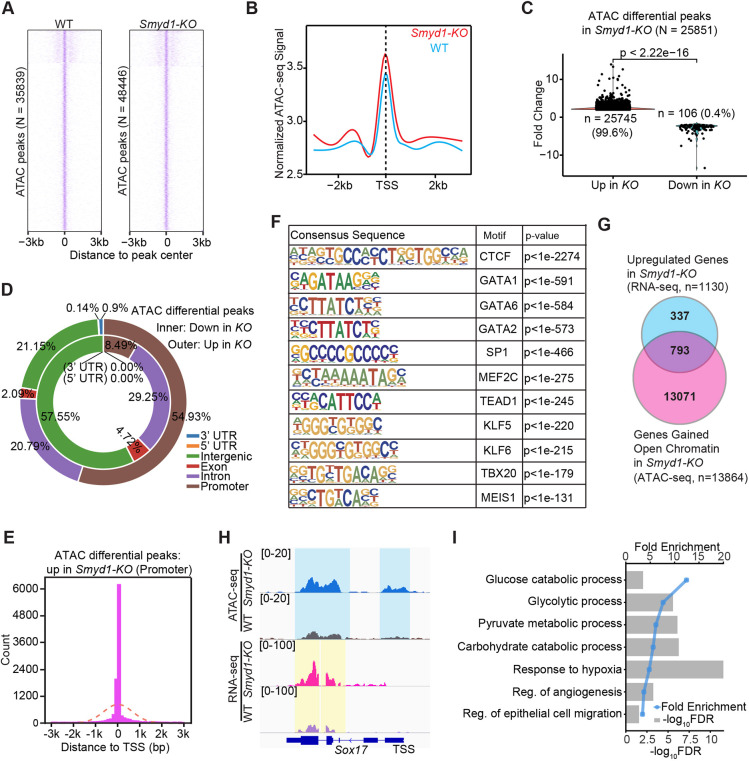
**Chromatin accessibility of *Smyd1-KO* hearts.** (A) Heatmap of normalized ATAC-seq read counts of E9.5 WT and *Smyd1-KO* hearts, ranked by total intensity. Reads are centered on the middle of the accessible peak ±3 kb. White and purple are low and high signal intensity, respectively. (B) Metagenes plot of E9.5 WT and *Smyd1-KO* normalized ATAC-seq signal ±3 kb around the TSS of all identified loci. (C) Violin plot with individual dots showing differential ATAC peaks (*n*=25,851) in *Smyd1-KO* hearts (twofold change). (D) Two-layer donut chart showing the genomic distribution of the differential ATAC peaks in *Smyd1-KO* hearts. Inner circle shows peaks lost in *Smyd1-KO*; outer circle shows peaks gained in *Smyd1-KO*. (E) Histogram with the fitted line showing counts of gained peaks within 3 kb of TSS (90.5% of all peaks in the promoter regions). (F) Known consensus motifs enriched in ATAC-seq loci upregulated in *Smyd1-KO* hearts. (G) Venn diagram of overlapped genes that gained open chromatin by ATAC-seq (*n*=13,864) and upregulated genes (*n*=1130) in RNA-seq of *Smyd1-KO* hearts. (H) Representative ATAC-seq and RNA-seq Integrative Genomics Viewer (IGV) browser tracks of *Sox17*. (I) GO biological processes terms for overlapped genes from G. Bar length represents −log_10_(FDR), and the blue line represents fold enrichment of each GO term.

Chromatin accessibility at gene regulatory regions affects transcriptional output. To examine chromatin status in response to the absence of SMYD1, we performed ATAC-seq (Buenrostro et al., 2015; Corces et al., 2017) with E9.5 WT and *Smyd1-KO* hearts. We identified 35,839 and 48,446 transposase-accessible genomic loci in WT and *Smyd1-KO* hearts, respectively (Fig. 5A, Tables S6, S7). The number of loci with open regions in *Smyd1-KO* was ∼35% greater than that in WT hearts. Further, we found that the ATAC-seq signals at regions within 3 kb of transcription start sites (TSSs) were significantly higher in the knockout (Fig. 5B). Remarkably, our ATAC-seq analysis revealed that 99.6% of the 25,851 differential ATAC peaks were increased accessibility in *Smyd1-KO* hearts (twofold change), and only 106 lost accessibility in *Smyd1-KO* hearts (twofold change) (Fig. 5C). Genomic annotation further revealed that 54.93% of the 25,745 gained peaks in knockout hearts, but only 8.49% of the 106 lost peaks, were assigned to the promoter region (within 10 kb of the TSS) (Fig. 5D). Furthermore, 90.5% of the promoter regions with increased accessibility were proximal to the TSS (within 3 kb) (Fig. 5E). These results suggested that chromatin structure was robustly remodeled in the absence of SMYD1 in developing heart and that SMYD1 was more likely to close chromatin or, at least, limit chromatin accessibility to proximal gene promoters. Genomic loci with increased ATAC-seq peaks were greatly enriched for the DNA motif of core transcriptional regulators of endothelial cells (i.e. CTCF, GATA1, GATA6, GATA2, SP1, KLF5, TEAD1), which suggested that the SMYD1 remodels the chromatin in the context of these transcription regulators in the developing hearts (Fig. 5F).

We also identified 793 upregulated genes in *Smyd1-KO* hearts that showed open chromatin with *Smyd1* null (Fig. 5G). *Sox17* is an example of an overlapped differentially accessible gene (Fig. 5H); *Sox17* is required for endocardium development and arterial remodeling of coronary vasculature during heart development (Saba et al., 2019). By performing GO enrichment analysis with these 793 genes, glycolytic processes, response to hypoxia, and angiogenesis (Fig. 5I) again appeared as the top affected pathways.

In summary, the chromatin accessibility results indicate that SMYD1 restricts chromatin accessibility at proximal promoters, and SMYD1 restricts the accessibility of genes involved in metabolic and angiogenesis programs during embryonic heart development.

### Chromatin accessibility in *Chd4-CMko* hearts

**Fig. 6.**
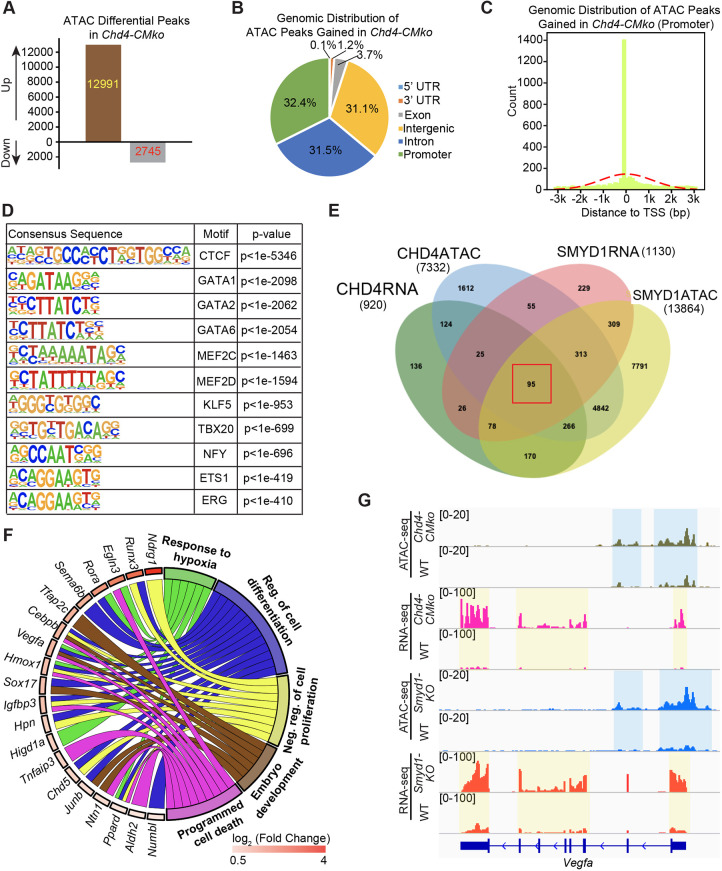
**CHD4 and SMYD1 repress a common subset of pathways.** (A) Numbers of differential ATAC-seq peaks in *Chd4-CMko* hearts. (B) Genomic distribution of locations in which ATAC peaks were gained in *Chd4-CMko* hearts. (C) In *Chd4-CMko* hearts, 73% of the differentially accessible peaks found in the promoter regions were within 3 kb of the TSS. (D) Known consensus motifs enriched in ATAC-seq loci upregulated in *Chd4-CMko* hearts. (E) Venn diagram of overlapping genes in indicated datasets. The red square highlights the 95 genes present in all four datasets. (F) The circular plot of representative genes of the 95 shared genes from E simultaneously presents a detailed view of the relationships between expression changes (left semicircle perimeter) and enriched biological processes (right semicircle perimeter). Color bar represents log_2_ fold change of gene expression originally from the RNA-seq differential gene expression analysis in *Chd4-CMko* hearts. (G) Representative CHD4 ChIP-seq in WT hearts (Wilczewski et al., 2018), ATAC-seq and RNA-seq Integrative Genomics Viewer (IGV) browser tracks of *Vegfa* of both *Smyd1-KO* and *Chd4-CMko*.

To further define the function of SMYD1–CHD4 interaction in remodeling chromatin accessibility, we performed ATAC-seq on E10.5 *Chd4-CMko* hearts. We identified 12,991 peaks that were gained (twofold change) and 2754 peaks lost (twofold change) in the knockout hearts (Fig. 6A, Tables S8, S9). These differentially accessible peaks were evenly located at intergenic regions (31.1%), introns (31.5%) and promoters (32.4%) (Fig. 6B), and 73% of the differentially accessible peaks were at promoter regions within 3 kb of TSSs (Fig. 6C). As with peaks that represented gained accessibility in *Smyd1-KO*, genomic loci with increased ATAC-seq signals in *Chd4-CMko* hearts were also enriched for DNA-binding motifs of CTCF, GATAs, MEF2C and KLF5 (Fig. 6D).

### SMYD1 and CHD4 repress a common subset of genes related to cell proliferation and response to hypoxia

Next, we systematically compared the upregulated gene lists of RNA-seq from *Chd4-CMko* and *Smyd1-KO* with genes associated with peaks gained in the ATAC-seq of the two mouse lines. We identified 95 genes at the intersection of the four datasets (Fig. 6E). These 95 genes were enriched in pathways of cellular response to hypoxia (e.g. *Vegfa*, *Hmox1*, *Rora*), and these genes were also related to negative regulation of cell proliferation and programmed cell death (e.g. *Igfbp3*, *Hmox1*, *Ppard*) (Fig. 6F,G). These data reveal that SMYD1 and CHD4 repress a common subset of gene programs and pathways in the developing heart.

## DISCUSSION

Chromatin remodeling and DNA accessibility are fundamental molecular events in the regulation of gene expression. Alteration of chromatin architecture is a directed process that requires large, multi-component complexes composed of core sets of enzymes. Proteins that configure a core remodeling complex, and hence conduct the enzymatic reactions, have been defined by *ex vivo* interaction studies, such as glutathione S-transferase pull downs or yeast two-hybrid assays (Dorr and Conlon, 2019; Slagle and Conlon, 2016). When intact epigenetic complexes have been isolated, investigators usually characterize the complexes in tissue culture cells or in a single tissue type (Dorr and Conlon, 2019). These approaches have limited capability to determine the temporal and spatial interactome under physiological conditions and the enzymatic activities executed by a given complex.

In addition to previously characterized components of the NuRD complex in the endogenous CHD4 cardiac interactome (Robbe et al., 2022), we discovered CHD4 in complex with the histone methylase SMYD1, and we defined this interaction by a variety of biochemical assays. Our transcriptomic and chromatin accessibility analyses of *Smyd1-KO* and *Chd4-CMko* embryonic hearts revealed that the SMYD1 and CHD4 repress common transcription programs and pathways, including those relating to glycolysis, response to hypoxia, and angiogenesis. Our study provides a new understanding of the function of CHD4 and SMYD1 in the regulation of heart development.

Investigators had previously established the interaction between SMYD1 and HDAC, components of the NuRD complex, and SMYD1 was considered to be an HDAC-dependent transcriptional repressor (Gottlieb et al., 2002). However, there was no evidence that the interaction between SMYD1 and HDAC was direct. Our SPR assay showed that SMYD1 interacts directly with another NuRD component, namely CHD4. It is likely that CHD4 functions as a bridge between SMYD1 and HDAC, but this remains to be determined by further biochemical assays.

By correlating differentially expressed genes identified in *Smyd1-KO* and *Chd4-CMko* embryonic hearts, we found that 76.7% of the shared differentially expressed genes were upregulated in the absence of SMYD1 or CHD4. This finding strongly suggested that SMYD1 and CHD4 are responsible for repressing a common subset of cardiac genes. Interestingly, one of the over-represented pathways for these shared upregulated genes was vasculature development (Fig. 4H). We observed a significant upregulation of *Vegfa*, a gene repressed by CHD4 and SMYD1, in the embryonic mouse hearts lacking *Chd4* or *Smyd1*. This finding aligns with prior research indicating the crucial role of myocardial VEGFA-endothelial VEGFR2 signaling in coronary angiogenesis and arterial formation during heart development (Wu et al., 2012). Given our specific knockout of *Chd4* in cardiomyocytes and the predominant expression of *Smyd1* in cardiomyocytes, we propose that deficiency in CHD4 and SMYD1 within cardiomyocytes leads to an enrichment of the vasculature development pathway, facilitated by the upregulated myocardial VEGFA signaling. Investigating the epigenetic regulation of the cardiomyocyte paracrine pathway, mediated by CHD4 and SMYD1, during embryonic heart development and adult heart homeostasis will be of significance for a comprehensive understanding of their roles in cardiac physiology.

We observed downregulation of 398 and 626 genes in *Chd4-CMko* and *Smyd1-KO* hearts, respectively, suggesting that both CHD4 and SMYD1 may directly or indirectly activate gene transcription during heart development. Given that SMYD1 trimethylates histone H3K4 (H3K4me3) (Wang et al., 2021; Warren et al., 2018), a modification associated with open chromatin, we speculate that genomic loci abundant in H3K4me3 binding may coincide with SMYD1 binding. To investigate this, SMYD1 chromatin immunoprecipitation with sequencing (ChIP-seq) analyses would be beneficial, as neither RNA-seq nor ATAC-seq can directly demonstrate the regulatory effects of proteins on transcription. However, despite our attempts and those of others (Franklin et al., 2011), we were unable to obtain sufficient endogenous SMYD1 for DNA sequencing. ChIP-seq experiments using two commercial and one homemade SMYD1 antibody on cardiac and skeletal muscles, as well as SMYD1-transfected HEK-293 cells, were unsuccessful. Consequently, the absence of ChIP-grade SMYD1 antibodies has hindered our identification of direct SMYD1-bound genomic loci in the heart through genomic sequencing. If SMYD1-ChIP-seq data from embryonic hearts becomes available, overlapping it with H3K4me3-, CHD4- and HDAC-ChIP data could help identify whether CHD4 and SMYD1 colocalize at open chromatin sites devoid of HDAC binding. Genes associated with these sites may correspond to the downregulated genes identified in *Smyd1-KO* and *Chd4-CMko* hearts. Additionally, this overlapping analysis might reveal whether CHD4 and SMYD1 can open chromatin independently of the HDAC/NuRD complex or whether CHD4 and SMYD1 work independently of each other during heart development.

The other limitation of our current study is that we have not determined the genetic interaction of SMYD1 and CHD4 in the developing hearts. For instance, mice carrying heterozygous *Chd4* null alleles in cardiomyocytes (*Tnnt2^Cre/+^;Chd4*^flox/+^) and *Smyd1^+/−^* mice exhibit typical heart development and survival rates. However, the cardiac phenotype of embryos with compound heterozygous null alleles for *Chd4* and *Smyd1* (*Tnnt2^Cre/+^;Chd4*^flox/+^*;Smyd1^+/−^*), particularly if they die as a result of cardiac defects at the same developmental stage (E10.5), is yet to be elucidated.

In summary, this study provides a comprehensive exploration of the SMYD1 and CHD4 cardiac interactomes in embryonic mouse hearts, alongside transcriptomic and chromatin accessibility datasets for *Smyd1-KO* and *Chd4-CMko* embryonic hearts. These findings could serve as valuable resources for studying the epigenetic regulation of heart development.

## MATERIALS AND METHODS

### Mouse and cell lines

The Institutional Animal Care and Use Committee at the University of North Carolina approved all murine experiments, which were performed according to the Committee guidelines. *Chd4* conditional knockout (*Chd4-CMko*) mice and control littermates were produced as described (Wilczewski et al., 2018). WT and *Smyd1^+/−^* (strain #006473) mice were obtained from the Jackson Laboratory. Both male and female mice/embryos were used unless specified. The HEK-293 cell line was obtained from ATCC (CRL-1573), and their contamination status was checked at regular intervals.

### Preparation of cardiac nuclei

Frozen hearts from 6-week-old female WT CD1 mice were homogenized using a mortar and pestle in liquid nitrogen. Nuclei were prepared as described (Franklin et al., 2011) and snap-frozen in liquid N_2_.

### Solubilization of protein complexes

Plasmids were transfected into HEK-293 cells, and cells were harvested as described (Shi et al., 2021). E10.5 WT CD1 hearts (minimum 28 hearts per IP, three separate biological replicates) were harvested in cold PBS. Harvested cells, embryonic hearts, or prepared cardiac nuclei were snap-frozen and cryolysed as described (Waldron et al., 2016). Frozen tissue powder was resuspended in optimized lysis buffer [5 ml/g powder; 20 mM K-HEPES pH 7.4, 0.11 M potassium acetate, 2 mM MgCl_2_, 0.1% Tween 20, 1 μM ZnCl_2_, 1 mM CaCl_2_, 0.5% Triton X-100, 150 mM NaCl, and protease and phosphatase inhibitors (Sigma-Aldrich)]. Samples were homogenized using a POLYTRON homogenizer (Kinematica) and processed for IP as described (Kaltenbrun et al., 2013; Kennedy et al., 2017; Robbe et al., 2022; Waldron et al., 2016).

### IP

For embryonic hearts and cardiac nuclei samples, conjugation of rabbit anti-CHD4 (Abcam, ab72418), rabbit anti-SMYD1 (Covance, NC867, generated by the F.L.C. lab) or negative control custom rabbit anti-IgG (Sigma-Aldrich, I5006) antibodies to magnetic beads (Invitrogen) was performed as described (Kaltenbrun et al., 2013; Kennedy et al., 2017; Waldron et al., 2016). For transfected HEK-293 cells, co-IP was performed using anti-TurboGFP magnetic beads (Chromotek, tbtma-100), anti-V5 magnetic beads (MBL International, M167-11) or anti-Flag magnetic beads (Origene, TA150042) with elution buffer at 95°C for 10 min. The immuno-isolated proteins were subjected to immunoblotting or MS analysis.

### Immunoblotting assay

Blots were blocked with 5% milk/TBST (Tris-buffered saline with 0.1% Tween 20) at room temperature for 1 h and then probed with the following primary antibodies, diluted in 10% blocking buffer (1% heat-inactivated goat serum and 0.1% Triton X-100 in PBS), overnight at 4°C: rabbit anti-Myc tag HRP (1:2500; Abcam, ab1326), rabbit anti-V5 (1:1000; Sigma-Aldrich, V8137), mouse anti-TurboGFP (1:1000; Origene, TA150041), rabbit anti-CHD4 (1:500; Active Motif, 39289), rabbit anti-SMYD1 (1:1000; Abcam, ab181372). After rinsing, blots were incubated in the following secondary antibodies, diluted in 10% blocking buffer, for 1 h at ambient temperature: mouse anti-rabbit IgG, light chain-specific HRP-conjugated antibody (1:10,000, Jackson ImmunoResearch, 211-032-171) or goat anti-mouse IgG, light chain HRP-conjugated antibody (1:10,000, Jackson ImmunoResearch, 115-035-174). Antibody–antigen complexes were visualized using an ECL Western Blotting Analysis System (Amersham).

### Proteomic analysis of CHD4 affinity purifications

The methodology employed for MS followed the protocol outlined by Shi et al. (2023). Initially, immuno-isolated proteins were separated using SDS-PAGE with a resolution of approximately 1.5 cm and then visualized through Coomassie Blue staining. Subsequently, each lane underwent in-gel digestion with trypsin and was analyzed using nano-liquid chromatography coupled to tandem mass spectrometry, which was consistent with established procedures. Tandem mass spectra were extracted using Proteome Discoverer (version 1.4, Thermo Fisher Scientific) and queried against a theoretical tryptic peptide database derived from both forward and reverse entries of the mouse UniProt-SwissProt protein sequence database, along with common contaminants, employing the SEQUEST algorithm. The resulting SEQUEST search outcomes were assessed using Scaffold (version 4.6.1, Proteome Software, Inc.) with the local false discovery rate (LFDR) scoring approach to determine peptide and protein probabilities. Thresholds for peptide and protein probabilities were chosen to maintain a ≤1% false discovery rate (FDR) at the peptide level based on LFDR modeling and at the protein level, considering the number of proteins identified as matches to the reverse database. Proteins meeting these criteria and exhibiting a minimum of two unique peptides were selected, and their spectral counts were exported to Excel for further data analysis.

### SPR

SPR analysis was conducted by Profacgen, USA. Various concentrations of mouse SMYD1 peptides dissolved in water were manually deposited onto bare gold-coated PlexArray Nanocapture Sensor Chips (Plexera Bioscience) with a thickness of 47 nm, under conditions of 40% humidity. Replicate printing was performed for each concentration, with each spot containing 0.2 µl of the ligand solution. Subsequently, the chips were incubated overnight at 4°C under 80% humidity, followed by rinsing with 10× PBST (PBS with 0.1% Tween 20) for 10 min, 1× PBST for 10 min, and deionized water twice for 10 min. The chips were then blocked overnight with 5% (w/v) non-fat milk in water, followed by washing with 10× PBST for 10 min, 1× PBST for 10 min, and deionized water twice for 10 min. Prior to use, the chips were dried under a nitrogen stream.

SPR measurements were conducted using the PlexArray HT instrument (Plexera Bioscience). Collimated light at a wavelength of 660 nm passed through the coupling prism, was reflected off the SPR-active gold surface, and detected by a CCD camera. Buffers and samples were injected into the 30 μl flow cell mounted on the coupling prism using a non-pulsatile piston pump. Each measurement cycle comprised four steps: initial washing with running buffer at a constant rate of 2 μl/s to establish a stable baseline, sample injection at 5 μl/s for binding, surface washing with buffer at 2 μl/s for 300 s, and regeneration with 0.5% (v/v) H_3_PO_4_ at 2 μl/s for 300 s. All measurements were conducted at 4°C. Signal changes post-binding and washing (in arbitrary units) were recorded as assay values. Regions of interest corresponding to protein-grafted areas in the SPR images were analyzed, and the average reflectivity changes of these selected regions over time were plotted. Real-time binding signals were recorded and analyzed using the Data Analysis Module (DAM, Plexera Bioscience). Kinetic analysis was performed using Plexera Analysis software.

### Cardiomyocyte isolation

Isolation of embryonic mouse hearts was performed as previously described (Shi et al., 2023). Briefly, at E12.5, time-mated female WT CD1 mice were dissected, and ten embryonic hearts were collected and pooled into tubes. Sequentially, the hearts were rinsed with pre-warmed PBS, followed by culture media. Each tube received 1 ml of trypsin-EDTA (0.05%) for digestion at 37°C for 10 min. After discarding the solution from this initial incubation, fresh trypsin-EDTA (0.05%) was added (1 ml per tube), and hearts were homogenized using #19G needles. Subsequently, they were rotated at 37°C for another 10 min. Following this, the solution (excluding tissues) was transferred to a new tube, and trypsinization was repeated five times. All solutions containing dissociated cells were combined and centrifuged at 1000 ***g*** for 10 min at room temperature. The supernatant was aspirated, and the cell pellet was resuspended in 3 ml of culture media. The resuspended cells were seeded in a 6-cm dish for 1 h, allowing non-cardiomyocytes to attach to the plate while most cardiomyocytes remained in the culture media. Then, the cardiomyocytes and non-cardiomyocytes were seeded onto circular coverslips that were placed in a 12-well cell plate; 48 h later, the cells were fixed for PLA.

### PLA

Transfected HEK-293 cells or isolated cardiomyocytes were seeded onto circular coverslips and allowed to adhere for 24 h. Following this, the cells were fixed using 4% paraformaldehyde in PBS, permeabilized, and subsequently blocked with a blocking buffer (consisting of 10% heat-inactivated goat serum and 1% Triton X-100 in PBS) at room temperature for 1 h. The cells were then incubated overnight at 4°C with two specific primary antibodies, diluted in 10% blocking buffer, raised in different species: mouse anti-CHD4 (1:250; EMD Millipore, MABE455) and rabbit anti-SMYD1 (1:250; Abcam, ab34472). Negative controls included either no primary antibody or the use of only one of the primary antibodies.

Following rinsing steps, the cells were sequentially incubated with PLA probes for 1 h at 37°C, with ligase for 30 min at 37°C, and with polymerase for 100 min at 37°C, utilizing the Duolink^®^ In Situ Red Starter Kit Mouse/Rabbit (Sigma-Aldrich, DUO92101-1KT). For cardiomyocytes, subsequent to the PLA incubation, an anti-tropomyosin antibody (1:50; Developmental Studies Hybridoma Bank, CH-1), diluted in 10% blocking buffer, was applied overnight at 4°C, followed by PBST washes and incubation with goat anti-mouse Alexa Fluor 488-conjugated secondary antibody (1:1000; Thermo Fisher Scientific, A11001) diluted in 10% blocking buffer. After a final wash, the slides were mounted using the PLA mounting medium containing DAPI. Imaging was conducted using a Zeiss LSM 700 laser-scanning confocal microscope with a 63× oil objective.

### Immunohistochemistry

Immunohistochemistry was performed as previously described (Shi et al., 2023). Briefly, embryos at E9.5 (from female *Smyd1^+/−^* crossed with male *Smyd1^+/−^*) or at E10.5 (from female *Chd4^fl/fl^* crossed with male *Tnnt2^Cre/+^*; *Chd4^fl/+^*) were dissected and fixed in 4% paraformaldehyde in PBST at 4°C overnight. Following fixation, tissues were dehydrated, embedded in paraffin, and sectioned at a thickness of 10 μm. Sections were then de-waxed, rehydrated, and subjected to antigen retrieval by boiling in antigen retrieval buffer (10 mM sodium citrate, 0.05% Tween 20, pH 6.0) for 15 min. Subsequently, sections were blocked with 10% normal goat serum (NGS) in PBST at room temperature for 1 h. Primary antibodies against VEGFA (1:50; Thermo Fisher Scientific, MA5-13182) and cardiac troponin T (1:200; Abcam, ab209813), diluted in 10% blocking buffer, were applied and incubated overnight at 4°C. After washing three times with 1% NGS/PBST buffer, sections were incubated with a mixture of goat anti-mouse Alexa Fluor 488-conjugated secondary antibody (1:1000; Thermo Fisher Scientific, A11001) and goat anti-rabbit Alexa Fluor 546-conjugated secondary antibody (1:1000; Thermo Fisher Scientific, A11035), diluted in 10% blocking buffer, at room temperature for 1 h. Following three additional washes with 1% NGS/PBST buffer, slides were mounted with DAPI-containing mounting media. Imaging was performed using a Zeiss LSM 700 laser-scanning confocal microscope.

### DNA constructs

The complete coding sequence of human *CHD4*, tagged at the C terminus with Flag/Myc, was acquired from Origene (RC224232). To generate full-length turboGFP-tagged human SMYD1, a segment of cDNA reverse transcribed from human heart RNA (Takara, 636532) served as the template. Amplification was carried out using specific primers (5′ primer: GCAAGCTTATGACAATAGGGAGAATGGAGAAC; 3′ primer: CGCTCGAGTTGCTTCTTGTGGAACAGAGCTG), followed by digestion and insertion into the pCMV6-AC-turboGFP vector (Origene, PS100010) with HindIII/XhoI restriction enzymes.

Subsequently, the turboGFP tag was removed from the pCMV6-AC-turboGFP-SMYD1 construct to obtain pCMV6-AC-SMYD1, utilizing the Q5 site-directed mutagenesis kit (NEB, E0552S). For the full-length V5-tagged mouse Smyd1 construct, mouse heart cDNA was utilized as the template for amplification using specific primers (5′ primer: GACTAAGCTTATGACAATAGGCAGCATGGAG; 3′ primer: CAGTGATATCACACTGCTTCTTATGGAACAGAGCG). The resulting DNA fragment was digested with HindIII/EcoRV restriction enzymes and subsequently inserted into the pcDNA3.1 vector.

### RNA-seq

RNA-seq was performed with mouse embryonic hearts as described (Robbe et al., 2022; Wilczewski et al., 2018). Briefly, purified poly A RNA that had undergone two rounds of oligo-dT selection was converted into cDNA and used to generate RNA-seq libraries. Libraries were sequenced (Illumina HiSeq 2500, 75 bp paired-end reads) to a target depth of >30 million reads. Reads were aligned to the mm10 reference genome using STAR via the bcbio-nextgen RNA-sequencing pipeline. RNA-seq analysis was performed using DESeq2 (DESeq2_1.18.1) in R (4.3.2). A >0.5 log_2_ fold change, and adjusted *P*< 0.05 were considered statistically significant.

### ATAC-seq

Each embryonic mouse heart was dissected in cold PBS and individually cryopreserved in 200 µl of DMEM medium with 10% fetal bovine serum and 10% DMSO (v/v) using a Mr. Frosty isopropyl alcohol chamber (Skene et al., 2018). Genotyping was performed on individual samples to identify hearts with desired genotypes. Hearts from each genotype were thawed in a 37°C water bath and pooled (*n*=3 for E10.5, *n*=4 for E9.5 hearts per genotype per replicate). Samples were pelleted at 1000 ***g*** for 3 min at 4°C. The medium was aspirated, and the hearts were washed with cold PBS. Hearts were then dissociated in 200 µl of 0.05% Trypsin/EDTA at 37°C for 10 min, with gentle agitation every 3 min. Dissociation was stopped by adding 400 µl of DMEM/10% fetal bovine serum (v/v), and the samples were passed through a 100-μm cell strainer to remove the connective tissue (Li et al., 2019). Cells were quantified using Trypan Blue, and 40,000 viable cells were taken for downstream procedures. Dissociated embryonic cardiac cells were lysed to isolate nuclei, which were treated with Tn5 transposase (Nextera DNA Sample Prep Kit, Illumina) to isolate DNA (Buenrostro et al., 2015; Corces et al., 2017). Fragmented DNA was then amplified using bar-coded PCR primers, and libraries were pooled. Pooled libraries were then sequenced (Illumina Next-seq) to a depth of 100 million reads per sample. Reads were aligned to the mm10 reference genomes using BWA-MEM, and peaks were called using HOMERv4.10.3 (Heinz et al., 2010). A twofold change was set as the cutoff of differential changes. Annotation and analysis were performed using ChIP-Seeker v1.20.0 (Yu et al., 2015).

### GO analysis

GO terms were analyzed for proteins shared by SMYD1 and CHD4 embryonic cardiac interactomes using PANTHER (v18.0) Biological Process, Cellular Component, and Molecular Function using Fisher's Exact with FDR multiple test correction. PANTHER Biological Process (Fisher's Exact with FDR multiple test correction) terms were determined for differential genes from RNA-seq assays. Morpheus was used for heatmap visualization (http://software.broadinstitute.org/morpheus/). The enrichment circle in Fig. 2B and the raincloud plot in Fig. 4A were generated using the online tool SRplot (https://www.bioinformatics.com.cn/srplot).

### Statistical methods

The data are expressed as mean±s.e.m., and the number of independent biological and technical replicates is indicated in the legend of each figure. Welch's *t*-test or a parametric one-way ANOVA was employed for comparisons involving two or more two groups (followed by Tukey's posthoc test), respectively. *P*<0.05 was considered as significant: *P<0.05; **P<0.01; ***P<0.001; ****P<0.0001.

## Supplementary Material



10.1242/develop.202505_sup1 Supplementary information

Table S1. Representative CHD4 embryonic cardiac interacting proteins.

Table S2. Representative SMYD1 embryonic cardiac interacting proteins.

Table S3. Full list of CHD4 and SMYD1 embryonic cardiac interactomes.

Table S4. Differential genes in E9.5 *Smyd1-KO* mouse hearts.

Table S5. Shared Differential Genes Between E10.5 *Chd4-CMko* and E9.5 *Smyd1-KO*.

Table S6. Gained Peaks in E9.5 *Smyd1-KO* ATAC-seq.

Table S7. Lost Peaks in E9.5 *Smyd1-KO* ATAC-seq.

Table S8. Annotated Gained Peaks in E10.5 *Chd4-CMko* hearts ATAC-seq.

Table S9. Annotated Lost Peaks in E10.5 *Chd4-CMko* hearts ATAC-seq.
