# Supplementary material for: CHD4 and SMYD1 repress common transcriptional programs in the developing heart
Source: Development. 2024 May 3;151(8):dev202505. doi: 10.1242/dev.202505 (PMC11112163; doi:10.1242/dev.202505)
Supplement: Supplementary information [file develop-151-202505-s1.pdf]

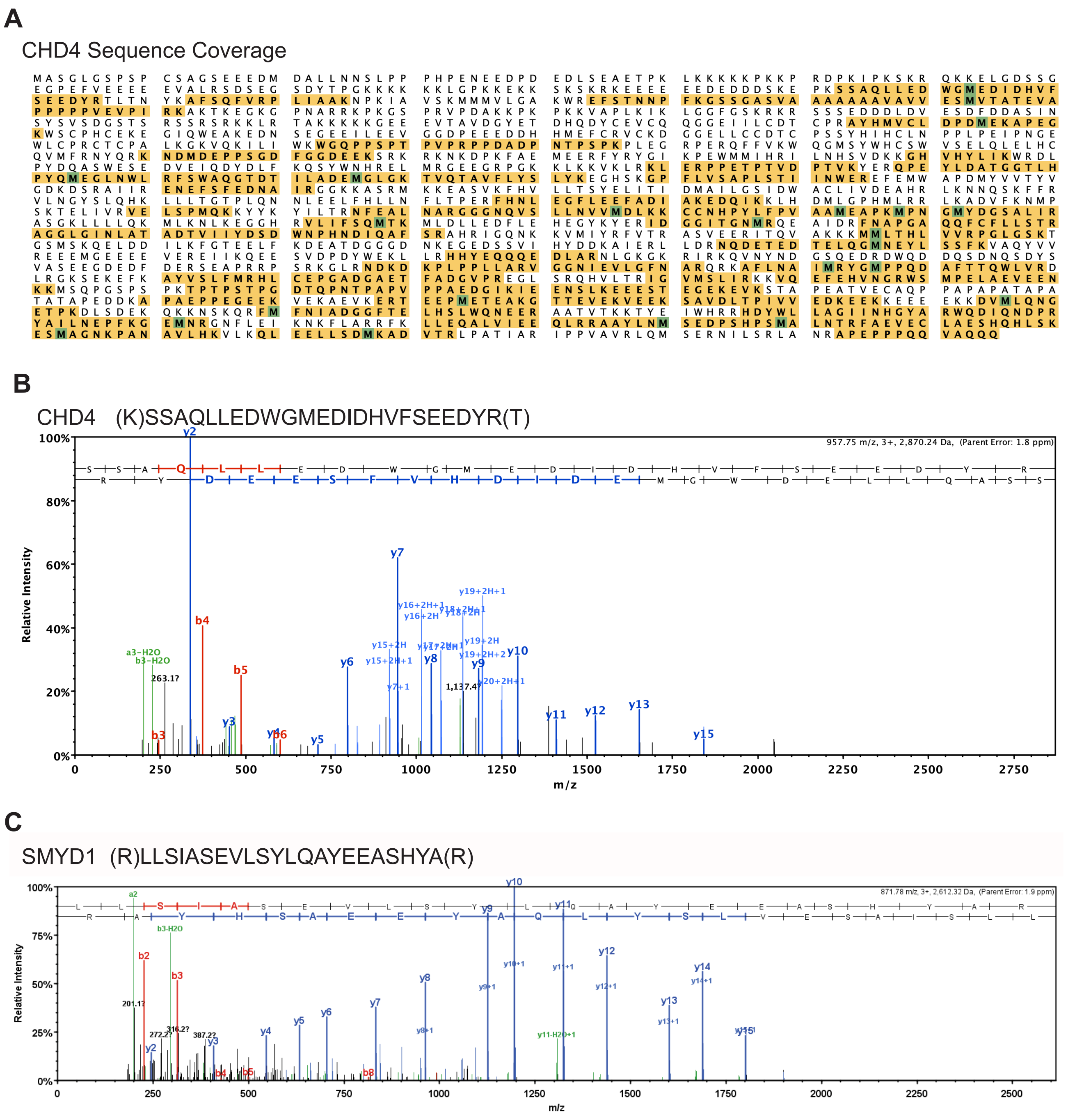

Fig. S1. Validation of the SMYD1-CHD4 interaction in murine heart tissue. (**A**) Sequence coverage of CHD4 from immune-isolates of E10.5 embryonic hearts (1100/1915 amino acids identified). (**B** and **C**) Identification of CHD4 (**B**) and SMYD1 (**C**) in immune-isolates of CHD4, as shown by CID MS/MS analysis of representative peptides.

Development • Supplementary information

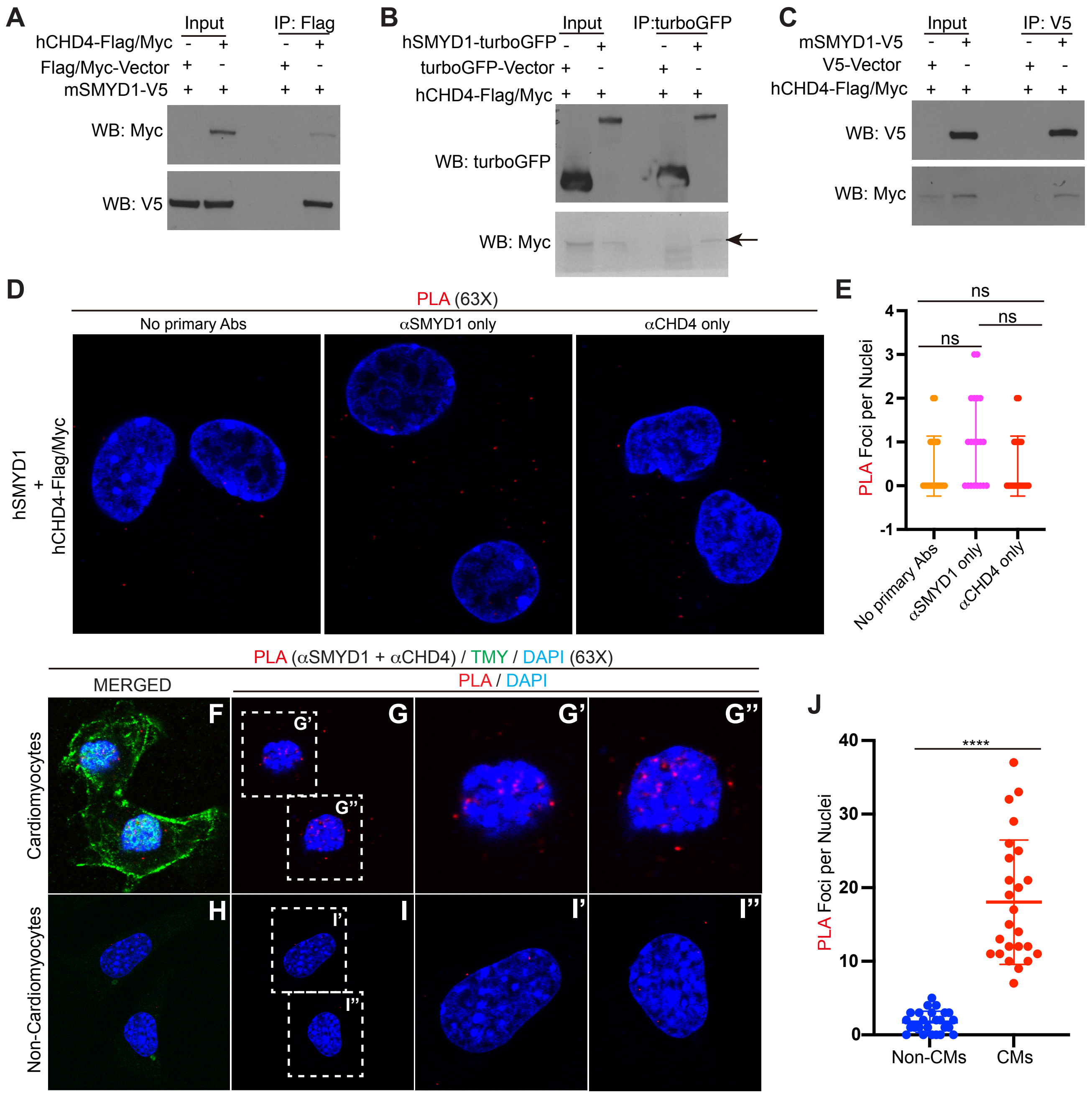

Development • Supplementary information

Fig. S2. CHD4 interacts with SMYD1. (**A**) Flag/Myc-tagged full-length human CHD4 (hCHD4-Flag/Myc) and V5-tagged full length mouse SMYD1 (mSMYD1-V5) are transfected into HEK-293 cells. Immunoprecipitation (IP) of exogenous CHD4 shows interaction with exogenous SMYD1. (**B**, **C**) Reciprocal IP confirms that CHD4 interacts with SMYD1. hCHD4-Flag/Myc and turboGFP-tagged full-length human SMYD1 (hSMYD1-turboGFP) (**B**) or mSMYD1-V5 (**C**) are transfected into HEK-293 cells. IP of exogenous human SMYD1 or mouse SMYD1 shows interaction with exogenous CHD4. (**A**-**C**) N=3-4 individual assays. (**D**) Constructs of Flag/Myc-tagged full-length human CHD4 and full-length human SMYD1 were transfected into HEK-293 cells. Proximity ligation assay (PLA) was performed using anti-SMYD1 or anti-CHD4 antibodies, or no primary antibody with the PLA assay kit, and the nuclei were stained with DAPI. (E) Quantification of the red foci in the nucleus in each condition. Data were obtained from 20 cells (from three independent replicates) per condition and are shown as the mean±s.e.m. ns: not significant ($P>0.05$, One-way ANOVA). (**F**-**I''**) PLA assay on isolated cardiomyocytes at E12.5. anti-Tropomyosin (TMY) was probed after the PLA ligation and amlification were completed. (**J**) Quantification of the red foci in the nucleus in each condition. Data were obtained from 25 cells (from two independent replicates) per condition and are shown as the mean±s.e.m. ****$P<0.0001$ (Welch's t-test).

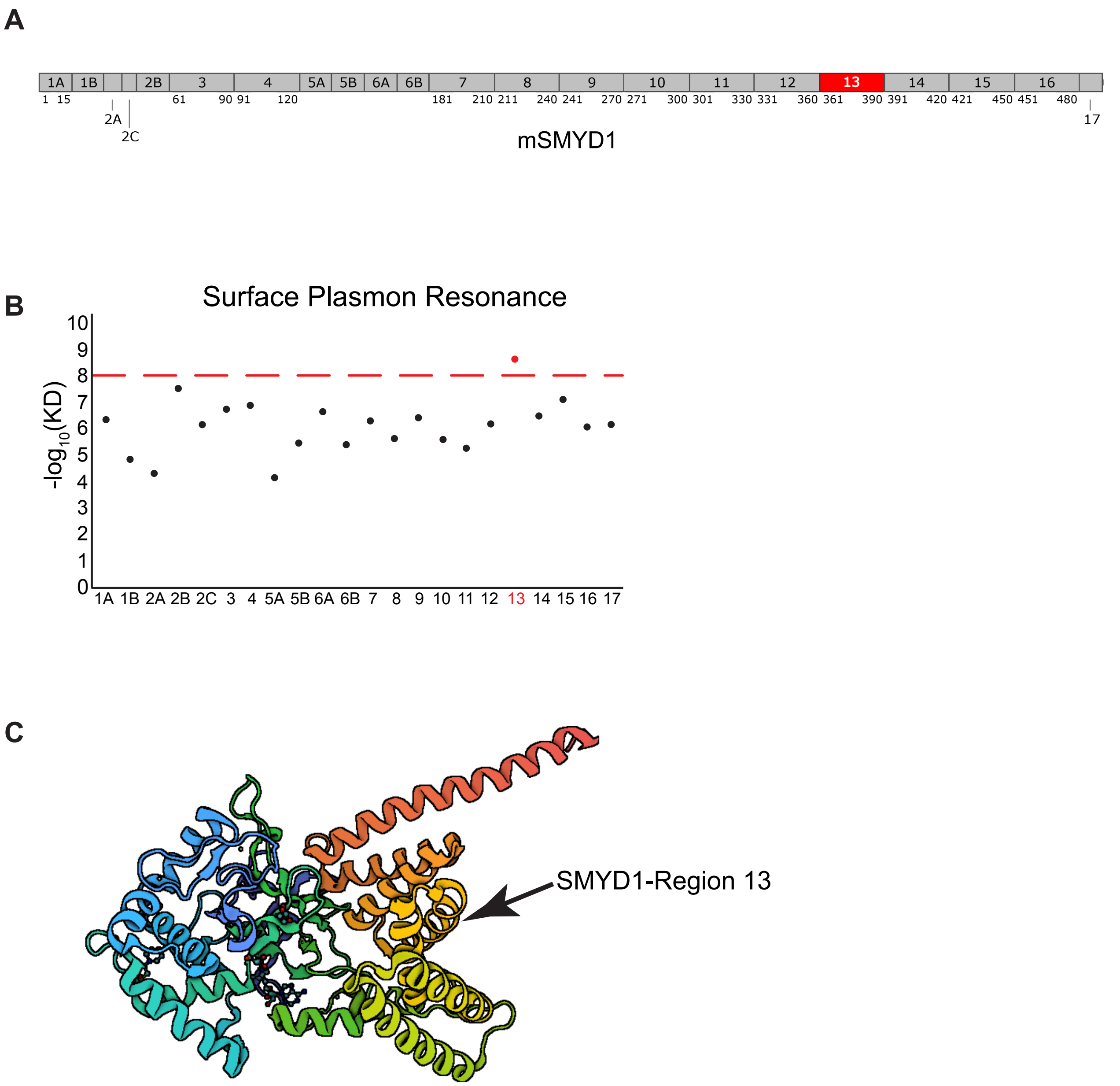

Fig. S3. Surface Plasmon Resonance assay of SMYD1-CHD4 interaction. (**A**) Schematic of mSMYD1 tiled peptides used in SPR assays. mSMYD1 was split into 22 peptides, and each was assayed for binding affinity to the carboxy—terminal portion of mCHD4. (**B**) Mouse SMYD1 interacts with CHD4 via surface plasmon resonance (SPR). Each dot represents the binding affinity of each SMYD1 peptide with CHD4. (**C**) Molecular structure modeling of mSMYD1 (PDB ID: 3N71). The SMYD1-Region13 is highlighted.

Development • Supplementary information

Table S1. Representative CHD4 embryonic cardiac interacting proteins.

Available for download at
https://journals.biologists.com/dev/article-lookup/doi/10.1242/dev.202505#supplementary-data

Table S2. Representative SMYD1 embryonic cardiac interacting proteins.

Available for download at
https://journals.biologists.com/dev/article-lookup/doi/10.1242/dev.202505#supplementary-data

Table S3. Full list of CHD4 and SMYD1 embryonic cardiac interactomes.

Available for download at
https://journals.biologists.com/dev/article-lookup/doi/10.1242/dev.202505#supplementary-data

Table S4. Differential genes in E9.5 *Smyd1-KO* mouse hearts.

Available for download at
https://journals.biologists.com/dev/article-lookup/doi/10.1242/dev.202505#supplementary-data

Table S5. Shared Differential Genes Between E10.5 *Chd4-CMko* and E9.5 *Smyd1-KO*.

Available for download at
https://journals.biologists.com/dev/article-lookup/doi/10.1242/dev.202505#supplementary-data

Table S6. Gained Peaks in E9.5 *Smyd1-KO* ATAC-seq.

Available for download at
https://journals.biologists.com/dev/article-lookup/doi/10.1242/dev.202505#supplementary-data

Table S7. Lost Peaks in E9.5 *Smyd1-KO* ATAC-seq.

Available for download at
https://journals.biologists.com/dev/article-lookup/doi/10.1242/dev.202505#supplementary-data

Table S8. Annotated Gained Peaks in E10.5 *Chd4-CMko* hearts ATAC-seq.

Available for download at
https://journals.biologists.com/dev/article-lookup/doi/10.1242/dev.202505#supplementary-data

Table S9. Annotated Lost Peaks in E10.5 *Chd4-CMko* hearts ATAC-seq.

Available for download at
https://journals.biologists.com/dev/article-lookup/doi/10.1242/dev.202505#supplementary-data
