# Supplementary material for: CHD4 and SMYD1 repress common transcriptional programs in the developing heart
Source: Development. 2024 May 3;151(8):dev202505. doi: 10.1242/dev.202505 (PMC11112163; doi:10.1242/dev.202505)
Supplement: Supplementary file 1 [file develop-151-202505_review_history.pdf]

CHD4 and SMYD1 repress common transcriptional programs in the developing heart

Wei Shi, Lauren K. Wasson, Kerry M. Dorr, Zachary L. Robbe, Caralynn M. Wilczewski, Austin J. Hepperla, Ian J. Davis, Christine E. Seidman, Jonathan G. Seidman and Frank L. Conlon
DOI: 10.1242/dev.202505

Editor: James Briscoe

Review timeline
Original submission: 3 November 2023
Editorial decision: 21 November 2023
First revision received: 11 March 2024
Accepted: 25 March 2024

Original submission

First decision letter

MS ID#: DEVELOP/2023/202505

MS TITLE: CHD4 and SMYD1 synergistically repress transcription in developing heart

AUTHORS: Wei Shi, Lauren K. Wasson, Kerry M. Dorr, Zachary L. Robbe, Caralynn M. Wilczewski, Austin J. Hepperla, Ian J. Davis, Christine Seidman, Jonathan Seidman, and Frank L Conlon

I have now received all the referees' reports on the above manuscript, and have reached a decision. The referees' comments are appended below, or you can access them online: please go to BenchPress and click on the 'Manuscripts with Decisions' queue in the Author Area.

As you will see, the referees express considerable interest in your work, but have some significant criticisms and recommend a substantial revision of your manuscript before we can consider publication. Most of the issues should be addressable by adding clarifications or adjusting the analysis of the data presented. Both referees comment on the PLA and this will probably need to be tackled by including additional data. I also agree with Referee 1 that biological or functional validation of any of the pathways of identified in the knockout hearts would strengthen the conclusions. If you are able to revise the manuscript along the lines suggested, which may involve further experiments, I will be happy receive a revised version of the manuscript. Your revised paper will be re-reviewed by one or more of the original referees, and acceptance of your manuscript will depend on your addressing satisfactorily the reviewers' major concerns. Please also note that Development will normally permit only one round of major revision. If it would be helpful, you are welcome to contact us to discuss your revision in greater detail. Please send us a point-by-point response indicating your plans for addressing the referees' comments, and we will look over this and provide further guidance.

Please attend to all of the reviewers' comments and ensure that you clearly highlight all changes made in the revised manuscript. Please avoid using 'Tracked changes' in Word files as these are lost in PDF conversion. I should be grateful if you would also provide a point-by-point response detailing how you have dealt with the points raised by the reviewers in the 'Response to Reviewers' box. If you do not agree with any of their criticisms or suggestions please explain clearly why this is so.

© 2024. Published by The Company of Biologists under the terms of the Creative Commons Attribution License (https://creativecommons.org/licenses/by/4.0/).

Reviewer 1

Advance summary and potential significance to field

This manuscript builds off the authors' previous publication in which they used IP-MS to define a protein interactome for the chromatin remodeling enzyme CHD4 in nuclei isolated from E10.5 mouse hearts (PMID: 35450884), a timepoint at which CHD4 is critical for cardiac development. One of the interesting interacting proteins from that screen was the histone methyltransferase SMYD1, which is also important for murine heart development at the same embryonic stage (PMID: 11923873). This manuscript focuses on validating the capacity for physical interaction between CHD4 and SMYD1 in vivo and in vitro. It also leverages new RNA-seq and ATAC-seq data generated from Smyd1-/- hearts and from cardiomyocyte-specific Chd4-deleted hearts to define overlapping pathways impacted by these gene deletions. The novelty of this study is that CHD4 and SMYD1 interactions have not been previously defined and that some overlapping gene pathways are upregulated in the hearts of each knockout (e.g., glycolysis, response to hypoxia, and angiogenesis). These findings may shed insights into novel mechanisms by which CHD4/SMYD1-containing complex promotes heart development. However, the authors should be cautious about overstating their conclusions since the pathway analyses gleaned from the transcriptomic/ATAC-seq data are currently correlative.

Comments for the author

1. Because there are no Re-ChIP (i.e., simultaneous occupancy) data for CHD4 and SMYD1 at any of the overlapping loci identified by the RNA-seq/ATAC-seq analyses, the word "synergistically" in the title of the paper seems like an overinterpretation. Similarly, the word "co-repress" in the title of Fig. 5 feels like too strong of a conclusion.
2. Some sort of biological or functional validation of any of the identified overlapping pathways of interest in the knockout hearts could strengthen the conclusions. For example, validation of upregulated protein expression for one of the key overlapping genes (i.e. VEGF-A or COL1A1) would be helpful especially since immunostaining might give additional spatial insights to the proposed SMYD1/CHD4 co-regulatory activity in the developing heart.
3. It is not clear from the proteomic and validation studies whether CHD4 and SMYD1 interact with each other in the context of the NuRD complex or independently. This is worth acknowledging in the text.
4. The cited reference by Ye et al (PMID: 26799706) indicates that SMYD1 is expressed in endothelial cells and promotes angiogenesis directly. However, the current manuscript states in at least two places that SMYD1 expression is restricted to cardiomyocytes. Please address this discrepancy, especially since global Smyd1-KO hearts were used for the transcriptomic analyses, and it is therefore unclear what cell type impacts the upregulated "regulation of vasculature development" pathway in these hearts.
5. For the PLA studies, how is it possible that PLA activity is detected in some cells without obvious expression of one of the interacting proteins of interest? For example, in Fig. 1G, PLA is detected in a cell that is negative for turboGFP (top left cell).
6. Please define how the GO analysis was performed in the methods.
7. Please cite data deposition and availability in the manuscript.
8. In Figs. 3 and 5 and throughout the manuscript, please use more precise nomenclature for the cardiomyocyte-specific Chd4-ko hearts. Consider defining them as Chd4-CMko hearts instead of Chd4-KO hearts, since the latter nomenclature implies a global knockout. This precision will help clarify that the comparative studies were performed on global Smyd1-KO hearts (Smyd1-/-) and on Chd4-CMko hearts.
9. Consider commenting on what happens to Smyd1 expression in Chd4-CMko hearts and what happens to Chd4 expression in Smyd1-/- hearts. These data should be available from the RNA-Seq data.
10. The figure labels for 4G are unclear; try to clarify that the data are showing an overlap between genes that gained open chromatin by ATAC-seq and upregulated genes by RNA-seq.
11. The figure legend for 5B is unclear; consider something like "genomic distribution of locations in which ATAC peaks were gained in Chd4-CMko hearts."
12. Better legends are needed for Table S1 to explain what the "overlapping" column means (since the proteins do not align in the first two columns).

© 2024. Published by The Company of Biologists under the terms of the Creative Commons Attribution License (https://creativecommons.org/licenses/by/4.0/).

13. The figure legend for 5C is confusing; it should probably say that 73% of the differentially accessible peaks found in promoter regions were within 3kb of the TSS.
14. For Fig. 5E, how is it possible that more genes are identified as overlapping in all 4 data sets than are defined as overlapping in 2 or 3 of the data sets?
15. The first sentence of p14 is inaccurate; the CHD4 interactome in developing mouse hearts was defined in the authors' previous study (PMID: 35450884) rather than in this one.
16. Please justify why the C-terminal portion of CHD4 was used for the SPR assay. Also, for S3C, it might be helpful to label the arrow as "SMYD1-region 13".

Reviewer 2

Advance summary and potential significance to field

Shi and colleagues investigate the role of CHD4 and SMYD1 in cardiac development. They demonstrate that the two proteins interact via co-IP and IP/MS approaches in a variety of cardiac tissues. They then go onto to compare transcriptomic and accessibility changes upon loss of these proteins identifying a commons set of changes. The data are helpful in helping understand how CHD4 and SMYD1 control cardiac development, and the datasets generated in this study will helpful to researchers in the cardiac development field.

Comments for the author

Overall, the manuscript is well-written and the figures are easy to follow. In general, while the manuscript is largely descriptive, the conclusions are well supported. I have a few suggestions for the authors to consider:
1. The co-IP in human iPSC-derived cardiomyocytes is a bit unclear and probably should be repeated. The band for Smyd1 upon CDH4 IP is very difficult to see and this is additionally compounded by the non-specific band in the IgG input lane. Similarly, in Fig. S2BC, the CHD4-myc band is a bit difficult to interpret since there appears to be a band at the molecular weight corresponding to CHD4-myc in the non-transfected control input lane.

2. A PCA of the individual RNA-seq replicates would be helpful as well as a heat map of the gene expression differences underlying the systems analyses presented in Fig 2B, 2C and shared between CHD4 and SMYD1 KO.

3. Additional pictures of the PLA would be helpful, specifically demonstrating the punctae representing the interaction is in the nucleus. A large fraction of the signal appears to be in the cytoplasm in the current images. Moreover, the authors should ensure they are not max projections and rather a single Z-plane (and hence something above or below the nucleus is not mis-interpreted to be in the nucleus.) It would be more powerful is the authors were able to perform it in a cell type expressing endogenous CHD4 and/or SMYD1.

4. It would be helpful to know whether CHD4 and SMYD1 are expressed in any cardiac cells (primarily endothelial cells) outside of the cardiac myocytes at E9.5. This would provide additional context to interpret the KO studies since the SMYD1 is a global KO. Additional characterization of its tissue specific expression pattern (whether in cardiac progenitors, cardiac mesoderm progenitors, etc) would be additionally helpful.
Perhaps RNA-scope of sections and/or mining of single cell datasets published in the field can quickly answer this question.

5. The discussion would benefit from additional text describing a few of the limitations of the study - including that it is unclear if these two factors are binding a particular genomic region at the same time whether their binding is interdependent and/or if their interaction is required for the transcriptional effects observed.

6. Given that CTCF is not a main point in the manuscript, I suggest toning down the mention of it. Also, I disagree that it is "the leading regulator for genome-wide organization of chromatin

© 2024. Published by The Company of Biologists under the terms of the Creative Commons Attribution License (https://creativecommons.org/licenses/by/4.0/).

architecture” (page 11). It is clearly important, but hard to argue that it is “the leading regulator”. This should be edited in the text.

Minor:
Abstract - “SMYD1 and CHD4 synergistically repress a group of genes and pathways that included glycolysis response to hypoxia, and angiogenesis “ - This is a bit of an overstatement, as the current data is in line with synergistic regulation of a set of genes, but does not conclusively demonstrate it. Hence, I would suggest toning this point down.

Discussion (final paragraph) - “In conclusion, the results of this study explain the mechanism by which CHD4 and SMYD1 function in heart development.” - this should be toned down.

The authors focus on the genes which are unregulated and gain accessbility upon SMYD1 or CHD4 deletion but a fraction of genes are down regulated. Could the authors comment/speculate on what they envision happening at these loci in the discussion?

First revision

Author response to reviewers' comments

Reviewer 1 Advance Summary and Potential Significance to Field:
This manuscrip builds off the authors’ previous publication in which they used IP-MS to define a protein interactome for the chromatin remodeling enzyme CHD4 in nuclei isolated from E10.5 mouse hearts (PMID: 35450884), a timepoint at which CHD4 is critical for cardiac development. One of the interesting interacting proteins from that screen was the histone methyltransferase SMYD1, which is also important for murine heart development at the same embryonic stage (PMID: 11923873). This manuscript focuses on validating the capacity for physical interaction between CHD4 and SMYD1 in vivo and in vitro. It also leverages new RNA-seq and ATAC-seq data generated from Smyd1-/- hearts and from cardiomyocyte-specific Chd4-deleted hearts to define overlapping pathways impacted by these gene deletions. The novelty of this study is that CHD4 and SMYD1 interactions have not been previously defined and that some overlapping gene pathways are upregulated in the hearts of each knockout (e.g., glycolysis, response to hypoxia, and angiogenesis). These findings may shed insights into novel mechanisms by which CHD4/SMYD1-containing complex promotes heart development. However, the authors should be cautious about overstating their conclusions since the pathway analyses gleaned from the transcriptomic/ATAC-seq data are currently correlative.

Reviewer 1 Comments for the Author:

1. Because there are no Re-ChIP (i.e., simultaneous occupancy) data for CHD4 and SMYD1 at any of the overlapping loci identified by the RNA-seq/ATAC-seq analyses, the word “synergistically” in the title of the paper seems like an overinterpretation. Similarly, the word “co-repress” in the title of Fig. 5 feels like too strong of a conclusion.
Response: We greatly appreciate the reviewer’s critical comments. We have revised the manuscript title to “CHD4 and SMYD1 repress common transcriptional programs in the developing heart”. The title of Fig.5 (currently Fig. 6 in the revised manuscript) has also been revised (Line 653).

2. Some sort of biological or functional validation of any of the identified overlapping pathways of interest in the knockout hearts could strengthen the conclusions. For example, validation of upregulated protein expression for one of the key overlapping genes (i.e. VEGF-A or COL1A1) would be helpful, especially since immunostaining might give additional spatial insights to the proposed SMYD1/CHD4 co-regulatory activity in the developing heart.
Response: Thanks for this important suggestion. We performed immunohistochemistry (IHC) staining with anti-VEGFA on the Chd4 and Smyd1 mutant hearts. Our IHC staining results showed that the protein expression of VEGFA is much higher in both Chd4 and Smyd1 mutant hearts (Fig. 4D-G’), which is consistent with the mRNA expression patterns shown by the RNA-seq data (Lines 228, 229).

© 2024. Published by The Company of Biologists under the terms of the Creative Commons Attribution License (https://creativecommons.org/licenses/by/4.0/).

3. It is not clear from the proteomic and validation studies whether CHD4 and SMYD1 interact with each other in the context of the NuRD complex or independently. This is worth acknowledging in the text.
Response: In Table S2, we have shown the SMYD1 cardiac interactome that includes all NuRD components (CHD4, MTA1/2/3, HDAC1/2, RBBP4/7, GATAD2A/B, MBD2/3). This has been incorporated in the manuscript (Lines 183-186).
4. The cited reference by Ye et al (PMID: 26799706) indicates that SMYD1 is expressed in endothelial cells and promotes angiogenesis directly. However, the current manuscript states in at least two places that SMYD1 expression is restricted to cardiomyocytes. Please address this discrepancy, especially since global Smyd1-KO hearts were used for the transcriptomic analyses, and it is therefore unclear what cell type impacts the upregulated "regulation of vasculature development" pathway in these hearts.
Response: We appreciate the reviewer's critical comments on the expression of SMYD1. We mined three independent single-cell datasets and identified that cardiomyocytes are the main cell type expressing SMYD1 in the heart (see below). Endothelial cells seem to express, but minimal, SMYD1 in the heart. To avoid the discrepancy, we have revised our text so that SMYD1 expression is restricted to striated muscles (Lines 40, 88) or "predominant expression of Smyd1 in cardiomyocytes (Lines 320, 321)". We also discussed how Smyd1 and Chd4 deficiency regulates vasculature development in the revised manuscript (Lines 316-326).

NOTE: Figure provided for reviewer has been removed. It was created using data from The Tabula Muris Consortium (2018). Single-cell transcriptomics of 20 mouse organs creates a *Tabula Muris*. *Nature* 562, 367-372. doi:10.1038/s41586-018-0590-4.

NOTE: Figure provided for reviewer has been removed. It was created using data from *The Human Protein Atlas* (*v23.0.proteinatlas.org, https://www.proteinatlas.org/ENSG00000115593-SMYD1/single+cell+type/heart+muscle*)

NOTE: Figure provided for reviewer has been removed. It was created using data from de Soysa, T. Y., Ranade, S. S., Okawa, S., Ravichandran, S., Huang, Y., Salunga, H. T., Schricker, A., Del Sol, A., Gifford, C. A. and Srivastava, D. (2019). Single-cell analysis of cardiogenesis reveals basis for organ-level developmental defects. *Nature* 572, 120-124. doi:10.1038/s41586-019-1414-x

5. For the PLA studies, how is it possible that PLA activity is detected in some cells without obvious expression of one of the interacting proteins of interest? For example, in Fig. 1G, PLA is detected in a cell that is negative for turboGFP (top left cell).
Response: We have repeated the PLA assay on new transfected 293 cells (hSMYD1 construct with no tGFP tag and CHD4-Flag/Myc construct), and mouse anti-CHD4 (1:250, EMD Millipore, MABE455) and rabbit anti-SMYD1 (1:250, Abcam, ab34472) antibodies and imaged them with higher magnification (Fig. 1F,G).

6. Please define how the GO analysis was performed in the methods.
Response: We added the methods for performing GO analysis in the revised Methods section (Lines 548-556).

7. Please cite data deposition and availability in the manuscript.
Response: The raw sequencing data have been deposited in the GEO data repository under specific accession numbers: GSE260692 (E9.5 Smyd1-KO RNA-seq), GSE260690 (E9.5 Smyd1-KO ATAC-seq), and GSE260689 (E10.5 Chd4-CMko ATAC-seq). E10.5 Chd4-CMko RNA-seq data was retrieved from GSE109012. Proteomics data are available in the supplemental materials (Lines 565-570).

8. In Figs. 3 and 5 and throughout the manuscript, please use more precise nomenclature for the cardiomyocyte-specific Chd4-ko hearts. Consider defining them as Chd4-CMko hearts instead of Chd4-KO hearts, since the latter nomenclature implies a global knockout. This precision will help clarify that the comparative studies were performed on global Smyd1-KO hearts (Smyd1-/-) and on Chd4-CMko hearts.

© 2024. Published by The Company of Biologists under the terms of the Creative Commons Attribution License (https://creativecommons.org/licenses/by/4.0/).

Response: We appreciate the reviewer's important suggestion. We have revised the nomenclature accordingly.

9. Consider commenting on what happens to Smyd1 expression in Chd4-CMko hearts and what happens to Chd4 expression in Smyd1-/- hearts. These data should be available from the RNA-Seq data.
Response: Based on our RNA-seq results, Smyd1 gene expression is significantly downregulated in Chd4-CMko hearts (Fig. 4C') and Chd4 gene expression trends to be downregulated in Smyd1-KO hearts but not significantly (Fig. 4C'') (Lines 223-226).

10. The figure labels for 4G are unclear; try to clarify that the data are showing an overlap between genes that gained open chromatin by ATAC-seq and upregulated genes by RNA- seq.
Response: We have revised the labels accordingly. Thanks for the suggestion!

11. The figure legend for 5B is unclear; consider something like "genomic distribution of locations in which ATAC peaks were gained in Chd4-CMko hearts."
Response: We have revised the figure legend accordingly. Thanks for the suggestion!

12. Better legends are needed for Table S1 to explain what the "overlapping" column means (since the proteins do not align in the first two columns).
Response: We have provided new Tables regarding the interactomes. Please see the new Tables S1-S3. Thanks for the reviewer's suggestion!

13. The figure legend for 5C is confusing; it should probably say that 73% of the differentially accessible peaks found in promoter regions were within 3kb of the TSS.
Response: We have revised the legend accordingly. Thanks for the suggestion!

14. For Fig. 5E, how is it possible that more genes are identified as overlapping in all 4 data sets than are defined as overlapping in 2 or 3 of the data sets?
Response: We have switched from the Upset plot to the Venn diagram to more clearly present the genesets overlapping (currently, Fig. 6E). Any overlapped area in the Venn diagram shows overlapped genes exclusively in indicated datasets.

15. The first sentence of p14 is inaccurate; the CHD4 interactome in developing mouse hearts was defined in the authors' previous study (PMID: 35450884) rather than in this one.
Response: We have revised the text in the manuscript (Lines 298, 299).

16. Please justify why the C-terminal portion of CHD4 was used for the SPR assay. Also, for S3C, it might be helpful to label the arrow as "SMYD1-region 13".
Response: Previous studies have demonstrated that the C-terminus of CHD4 is the main region responsible for protein interaction. For example, notable enrichment of NuRD subunits was seen with CHD4-C when compared to CHD4-N. The subunit of the ChAHP complex, ADNP, a transcription repressor, NAB2, also interacts with CHD4 through the C-terminus of CHD4 (PMIDs: 34231305, 16574654). Thus, the C-terminal portion of CHD4 was used for the SPR assay to examine if CHD4 interacts with SMYD1. We have incorporated this in the text (Lines 153-163). Also, according to the reviewer's suggestion, we have labeled "SMYD1-region 13" in Fig. S3C.

***** Reviewer 2 Advance Summary and Potential Significance to Field:
Shi and colleagues investigate the role of CHD4 and SMYD1 in cardiac development. They demonstrate that the two proteins interact via co-IP and IP/MS approaches in a variety of cardiac tissues. They then go onto to compare transcriptomic and accessibility changes upon loss of these proteins, identifying a commons set of changes. The data are helpful in helping understand how CHD4 and SMYD1 control cardiac development, and the datasets generated in this study will helpful to researchers in the cardiac development field.

Reviewer 2 Comments for the Author:
Overall, the manuscript is well-written, and the figures are easy to follow. In general, while the manuscript is largely descriptive, the conclusions are well supported. I have a few suggestions for the authors to consider:

© 2024. Published by The Company of Biologists under the terms of the Creative Commons Attribution License (https://creativecommons.org/licenses/by/4.0/).

1. The co-IP in human iPSC-derived cardiomyocytes is a bit unclear and probably should be repeated. The band for Smyd1 upon CDH4 IP is very difficult to see and this is additionally compounded by the non-specific band in the IgG input lane. Similarly, in Fig. S2BC, the CHD4-myc band is a bit difficult to interpret since there appears to be a band at the molecular weight corresponding to CHD4-myc in the non-transfected control input lane.
Response: We appreciate the reviewer's comments. We have removed the Co-IP results on iCMs from the revised figures.
We have re-labeled the panels for Fig. S2B and C to present each sample's input and IP lanes more clearly.

2. A PCA of the individual RNA-seq replicates would be helpful as well as a heat map of the gene expression differences underlying the systems analyses presented in Fig 2B, 2C and shared between CHD4 and SMYD1 KO.
Response: We have included the PCA for Smyd1-KO RNA-seq in Fig. 3A. Heatmaps are added in Fig. 3E and 4B, as suggested.

3. Additional pictures of the PLA would be helpful, specifically demonstrating the punctae representing the interaction in the nucleus. A large fraction of the signal appears to be in the cytoplasm in the current images. Moreover, the authors should ensure they are not max projections and rather a single Z-plane (and hence something above or below the nucleus is not mis-interpreted to be in the nucleus.) It would be more powerful is the authors were able to perform it in a cell type expressing endogenous CHD4 and/or SMYD1.
Response: For transfected 293 cells, we have repeated the PLA assay and imaged them with higher magnification (Fig. 1F,G, S2D,E) using mouse anti-CHD4 (1:250, EMD Millipore, MABE455) and rabbit anti-SMYD1 (1:250, Abcam, ab34472) antibodies.

We also performed a PLA assay, using mouse anti-CHD4 (1:250, EMD Millipore, MABE455) and rabbit anti-SMYD1 (1:250, Abcam, ab34472) antibodies, with isolated mouse embryonic cardiomyocytes (E12.5) with co-stained tropomyosin (a marker for cardiomyocytes). Tromyosion-negative cells were used as a control (Fig. S2F-J).

All these new exogenous and endogenous PLA assays showed that the majority of PLA puncta exist in the nucleus. However, we still observe sparse PLA puncta outside the nucleus. We infer that some newly translated CHD4 protein interacts with SMYD1 in the cytoplasm, considering SMYD1 is localized in both the nucleus and cytoplasm.

4. It would be helpful to know whether CHD4 and SMYD1 are expressed in any cardiac cells (primarily endothelial cells) outside of the cardiac myocytes at E9.5. This would provide additional context to interpret the KO studies since the SMYD1 is a global KO. Additional characterization of its tissue-specific expression pattern (whether in cardiac progenitors, cardiac mesoderm progenitors, etc) would be additionally helpful. Perhaps RNA-scope of sections and/or mining of single-cell datasets published in the field can quickly answer this question.
Response: We appreciate the reviewer's critical comments and valuable suggestions! Please see our response to Reviewer 1's point #4.

5. The discussion would benefit from additional text describing a few of the limitations of the study - including that it is unclear if these two factors are binding a particular genomic region at the same time, whether their binding is interdependent and/or if their interaction is required for the transcriptional effects observed.
Response: Thanks for the reviewer's suggestions. We have included some discussions about the limitations of the study (Lines 332-352).

6. Given that CTCF is not a main point in the manuscript, I suggest toning down the mention of it. Also, I disagree that it is "the leading regulator for genome-wide organization of chromatin architecture" (page 11). It is clearly important, but hard to argue that it is "the leading regulator". This should be edited in the text.
Response: We have removed the text involving CTCF in the revised manuscript.

Minor:

© 2024. Published by The Company of Biologists under the terms of the Creative Commons Attribution License (https://creativecommons.org/licenses/by/4.0/).

Abstract - "SMYD1 and CHD4 synergistically repress a group of genes and pathways that included glycolysis, response to hypoxia, and angiogenesis " - This is a bit of an overstatement, as the current data is in line with synergistic regulation of a set of genes, but does not conclusively demonstrate it. Hence, I would suggest toning this point down.
Response: We have revised the text accordingly (Line 43). Thanks for the suggestion!

Discussion (final paragraph) - "In conclusion, the results of this study explain the mechanism by which CHD4 and SMYD1 function in heart development." - this should be toned down.
Response: We have revised the text accordingly (Lines 353-355).

The authors focus on the genes which are upregulated and gain accessibility upon SMYD1 or CHD4 deletion, but a fraction of genes are down regulated. Could the authors comment/speculate on what they envision happening at these loci in the discussion?
Response: We have included a discussion on those downregulated genes in the revised manuscript (Lines 327-345). Thanks for the suggestions!

Second decision letter

MS ID#: DEVELOP/2023/202505

MS TITLE: CHD4 and SMYD1 repress common transcriptional programs in the developing heart

AUTHORS: Wei Shi, Lauren K. Wasson, Kerry M. Dorr, Zachary L. Robbe, Caralynn M. Wilczewski, Austin J. Hepperla, Ian J. Davis, Christine Seidman, Jonathan Seidman, and Frank L Conlon

I am happy to tell you that your manuscript has been accepted for publication in Development, pending our standard ethics checks.

Reviewer 1

Advance summary and potential significance to field

This manuscript builds off the authors' previous publication in which they used IP-MS to define a protein interactome for the chromatin remodeling enzyme CHD4 in nuclei isolated from E10.5 mouse hearts (PMID: 35450884), a timepoint at which CHD4 is critical for cardiac development. One of the interesting interacting proteins from that screen was the histone methyltransferase SMYD1, which is also important for murine heart development at the same embryonic stage (PMID: 11923873). This manuscript focuses on validating the capacity for physical interaction between CHD4 and SMYD1 in vivo and in vitro. It also leverages new RNA-seq and ATAC-seq data generated from Smyd1-/- hearts and from cardiomyocyte-specific Chd4-deleted hearts to define overlapping pathways impacted by these gene deletions. The novelty of this study is that CHD4 and SMYD1 interactions have not been previously defined and that some overlapping gene pathways are upregulated in the hearts of each knockout (e.g., glycolysis, response to hypoxia, and angiogenesis), with validation of VEGFA gene/protein regulation, specifically. These findings may shed insights into novel mechanisms by which CHD4/SMYD1-containing complex promotes heart development.

Comments for the author

I appreciate the authors' responses to my comments and their modifications to the manuscript. The new PLA data and VEGFA immunostaining are strong additions to the study.

Reviewer 2

Advance summary and potential significance to field

© 2024. Published by The Company of Biologists under the terms of the Creative Commons Attribution License (https://creativecommons.org/licenses/by/4.0/).

The authors have adequately addressed my concerns. I have no additional concerns. Congratulations to the authors!

Comments for the author

See above

© 2024. Published by The Company of Biologists under the terms of the Creative Commons Attribution License (https://creativecommons.org/licenses/by/4.0/).
